# Employing *Streptomyces enissocaesilis* as a sustainable cell factory for green synthesis of gold nanoparticles with multi-target anticancer activity against MCF-7 breast cancer cells

**DOI:** 10.1186/s12934-026-03113-8

**Published:** 2026-09-28

**Authors:** Maisra M. El-Bouseary, Sara Ahmed Mohammed Mahmoud Badr, Fatma Sonbol, Tarek El‑banna, Amr Tayel, Mohamed M. El-Zahed, Engy Elekhnawy

**Affiliations:** 1 Department of Microbiology and Immunology, Faculty of Pharmacy, Tanta University, Tanta, 31527 Egypt; 2 Department of Pharmaceutical Chemistry, Faculty of Pharmacy, Alsalam University, Tanta, 31511 Egypt; 3 Department of Chemistry, The American University in Cairo, AUC Avenue, New Cairo, 11835 Egypt; 4 Department of Botany and Microbiology, Faculty of Science, Damietta University, New Damietta, 34517 Egypt

**Keywords:** RT-qPCR, c-erbB-2, p53, 16S rRNA gene sequencing, Flow cytometry

## Abstract

**Background:**

Currently, there is growing interest in gold nanoparticles (AuNPs) to be employed in cancer nanomedicine. This study describes the green synthesis of AuNPs using an actinobacterial isolate identified as *Streptomyces enissocaesilis* via 16 S rRNA sequencing. Characterization of the nanoparticles was performed using various techniques including UV-Vis spectroscopy, FTIR, TEM, XRD, EDX, and zeta-potential analysis. To provide insight into the interaction of AuNPs with MCF-7 breast cancer cells, a variety of apoptosis and cell proliferation assays were performed. These assays included concentration-dependent cytotoxicity testing, cell-cycle analysis, an Annexin V-FITC/PI apoptosis analysis, AO/PI staining, a BrdU proliferation assay, and an RT-qPCR to evaluate the expression of c-erbB-2 and p53.

**Results:**

Characterization of the AuNPs revealed that they were ruby-red, exhibited a strong SPR signal at 528 nm, were between 22.73 and 35.61 nm in size, had a positive zeta potential at + 27 mV, and exhibited a faceted crystal cubic structure. In regard to MCF-7 cells, the concentration-dependent cytotoxicity assay showed a clear dose-dependent inhibition of cell proliferation, with growth inhibition increasing from 20.14% at 3.125 µg/mL to 98.33% at 100 µg/mL and a half-maximal inhibitory concentration (IC_50_) of 8.32 µg/mL, considerably lower than the 18.90 µg/mL obtained for normal PBMC cells under the same protocol. Cell cycle analysis showed that AuNPs caused almost complete cell cycle arrest in the G0/G1 phase (99.25%) of the cell cycle, while both the G2/M and S phases were completely depleted. BrdU incorporation was significantly inhibited in a concentration-dependent manner. Finally, RT-qPCR and Annexin V-FITC/PI apoptosis analysis showed that AuNPs induced apoptosis (50.36%) and significantly inhibited the expression of the oncogene c-erbB-2, and in turn, they induced tumor suppressive pathway. The docking studies revealed that the Cys-Au species bound to biologically significant areas of ERα and CDK2, possibly explaining the antiproliferative effects observed in MCF-7 human breast cancer cells.

**Conclusions:**

These findings indicate that AuNPs biosynthesized using *Streptomyces enissocaesilis* exhibit significant anticancer effects against MCF-7 cells. The effects appear to be induced by a combination of antiproliferative, pro-apoptotic, and cell-cycle- and gene-modulatory effects. These effects validate the promise of Actinobacteria-stabilized AuNPs as a green nanoplatform for the treatment of human breast cancer.

**Graphical Abstract:**

**Figure:**
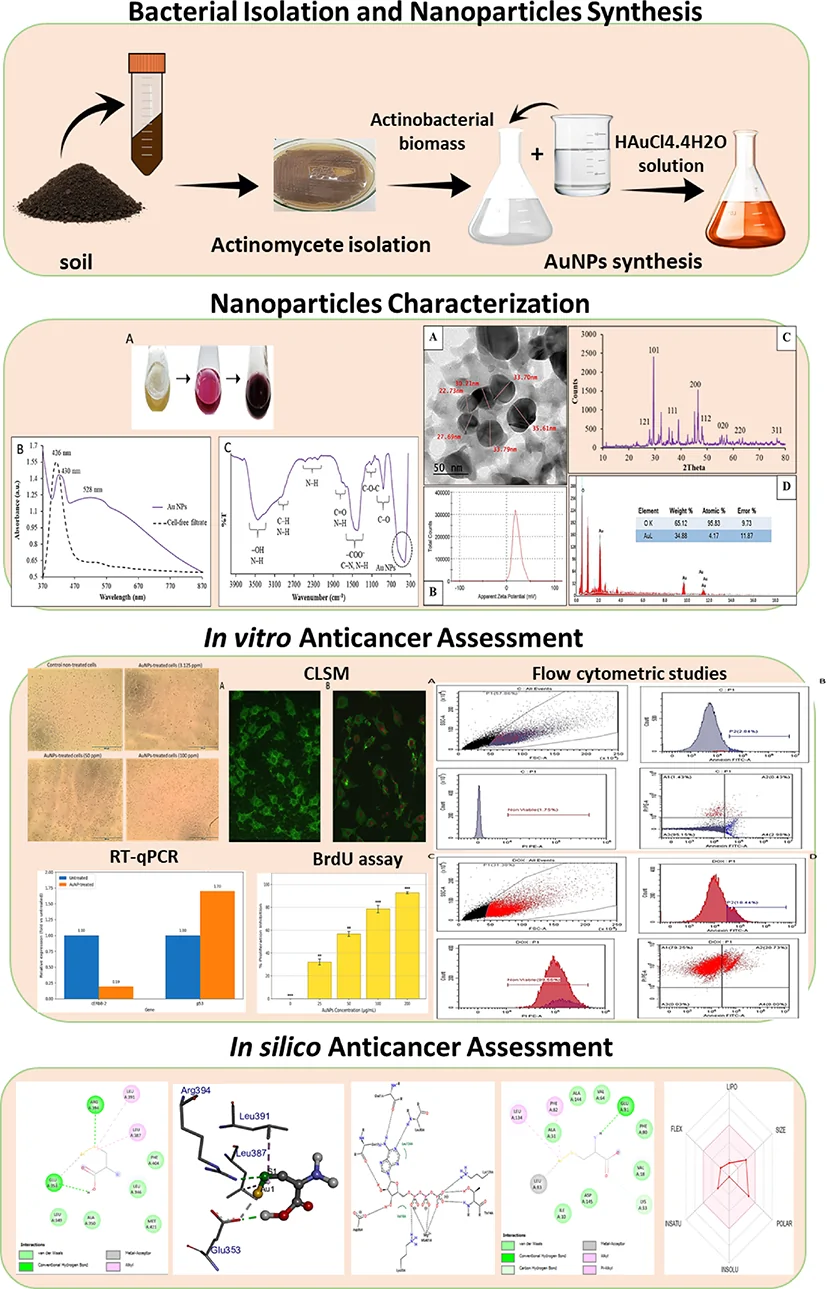

**Supplementary Information:**

The online version contains supplementary material available at https://doi.org/10.1186/s12934-026-03113-8.

## Background

Cancer remains a major global health challenge, and the three conventional treatment modalities, surgery, chemotherapy, and radiotherapy, each carry substantial limitations that restrict their long-term effectiveness. Surgery struggles to achieve complete tumor removal in heterogeneous or advanced disease, chemotherapy is limited by both the emergence of drug resistance and systemic toxicity to healthy tissue, and radiotherapy is constrained by precision issues and by side effects on surrounding normal tissue [1]. These combined limitations, together with the specific problem of acquired drug resistance, have driven sustained interest in alternative and complementary therapeutic strategies rather than in resistance-reversal approaches alone. The present study is concerned with developing one such alternative strategy rather than with directly overcoming drug resistance in existing chemotherapeutics.

Nanotechnology has become a key area of modern biomedical development as a result of nanoscale materials’ ability to interact with many different types of cells. Nanoparticles are of growing importance in biomedicine because their small size and high surface-area-to-volume ratio. Such properties allow them to cross biological barriers, achieve preferential accumulation in tumor tissue, and carry therapeutic or diagnostic payloads with a precision that is difficult to achieve with conventional small-molecule drugs [2]. Nanoparticle platforms have already found successful biomedical application in different areas, such as targeted drug delivery, in vivo imaging and diagnostics, and photothermal cancer therapy using plasmonic metal nanoparticles [2, 3]. Among metallic nanomaterials, gold nanoparticles (AuNPs) are the most attractive due to their excellent chemical stability, unique plasmonic behavior, and their versatility [1]. The field of oncology shows special interest towards AuNPs since they can serve as both as active nanosystems and as nanoscale drug delivery vehicles [1, 2].

Despite the advantages, AuNPs safety and therapeutic uses are highly dependent upon the synthesis route used. In traditional chemical synthesis, the use of harsh reducing agents, poorly biocompatible surfactants, and high-energy solvents is common [4]. This method often leaves behind toxic wastes and results in poorly biocompatible nanoparticles that are unsuitable for biomedical applications. These deficiencies have generated considerable interest towards the use of biological molecules in place of toxic reagents [3]. Green methods are preferred, not only for their safety, but also for producing nanoparticles with high biocompatibility [3, 4].

For green AuNP synthesis, microbial systems are especially attractive, since they secrete complex extracellular mixtures of enzymes, proteins, amino acids, pigments, and polysaccharides. In addition, they can be cultured at scale under mild, aqueous conditions more readily standardized than plant-extract-based synthesis, where phytochemical composition can vary with plant age, season, and geographic origin [5]. Such biomolecules are able to reduce Au³⁺ to Au⁰ and then cap the freshly formed metal core, which in turn governs colloidal stability, the aggregation state, and biological identity [5]. Actinomycetes are particularly valued here on account of their prolific secondary metabolism. Also, they have a well-documented capacity to release extracellular bioactive compounds, including antibiotics, hydrolytic enzymes, and redox-active pigments that can double as nanoparticle-reducing and capping agents [5, 6].

Breast cancer is a molecularly heterogeneous disease, classically subdivided into luminal A, luminal B, HER2-enriched, and triple-negative subtypes on the basis of estrogen receptor (ER), progesterone receptor (PR), and HER2 status. MCF-7 cells were selected for the present study on two independent, mechanistically relevant grounds. First, MCF-7 is an ER-positive, luminal-phenotype cell line, directly relevant to the ERα-docking arm of this study [2]. Second, and less commonly emphasized, MCF-7 retains wild-type, transcriptionally competent p53, in contrast to several other widely used breast cancer lines that carry loss-of-function p53 mutations; because p53 upregulation following AuNP treatment is a central endpoint of the present study, a p53-wild-type model is methodologically necessary, rather than merely convenient, for interpreting the RT-qPCR findings mechanistically.

Two molecular biomarkers were selected for expression analysis in this study: c-erbB-2 (HER2/ERBB2) and p53. c-erbB-2 is a receptor tyrosine kinase whose overexpression or amplification occurs in a clinically significant subset of breast cancers, driving proliferative and pro-survival signaling, correlating with a more aggressive clinical phenotype, and serving as a validated therapeutic target [3, 4]. p53, in contrast, is the central transcriptional regulator of cell-cycle checkpoints and apoptosis, and its functional loss is among the most common events in human cancer generally [5]. An agent capable of simultaneously suppressing c-erbB-2 and restoring or enhancing p53 activity would be expected to shift a cancer cell toward a less proliferative, more apoptosis-prone phenotype, motivating the combined measurement of both markers in the present study.

It is further evident from current work that how strongly AuNPs act against cancer is tied to their size, shape, surface charge, concentration, and the particular biological system they encounter. Certain biogenic AuNPs act mainly by reducing metabolic activity, whereas others act more broadly and trigger oxidative stress, mitochondrial depolarization, intrinsic apoptosis, autophagy, or cell-cycle arrest [1, 2]. Accordingly, the present study set out to bring microbiology together with cancer nanobiotechnology by employing an actinomycetes isolate to serve as a renewable nanofactory for producing eco-friendly AuNPs, after which their capacity to act against human breast cancer was assessed.

Several recent studies have reported the biosynthesis of AuNPs using *Streptomyces* species and evaluated their anticancer activity, including work with *Streptomyces albogriseolus* [6], *Streptomyces* sp. YJD18 [7], *Streptomyces* sp. ASM19 [8], and cancer-metabolite-derived AuNPs [9]. To the best of our knowledge, however, *Streptomyces enissocaesilis* has not previously been employed as a biological nanofactory for AuNP biosynthesis, and the present work is therefore the first report of its kind. Beyond the biosynthesis route itself, prior *Streptomyces*-AuNP anticancer studies have generally relied on one or two mechanistic endpoints, most often cytotoxicity paired with a single apoptosis or cell-cycle readout. The present study instead integrates six complementary, mechanistically layered endpoints, concentration-dependent cytotoxicity, cell-cycle analysis, Annexin V-FITC/PI apoptosis analysis, AO/PI confocal microscopy, BrdU proliferation analysis, and RT-qPCR expression of both an oncogene (c-erbB-2) and a tumor suppressor (p53), together with dual-target molecular docking against ERα and CDK2. This breadth of mechanistic triangulation, rather than the biosynthesis route alone, constitutes the principal contribution and the specific knowledge gap addressed by the present work.

## Materials and methods

### Isolation, purification, and cultivation of soil actinomycetes

Soil samples were collected from Tanta, Egypt, during July 2025. A total of 12 independent soil samples were collected from different locations across separate sampling points using simple agriculture tools. Soil specimens were taken at a depth of 5–15 cm, left to air-dry at room temperature over ne week, and subsequently heated dry at 60 °C for an hour. During isolation, a one-gram portion of the processed soil was dispersed in sterile physiological saline (0.85% NaCl) and taken through a serial dilution [10]. Portions drawn from the more dilute fractions were then spread onto starch casein agar (SCA) plates that had been enriched with nystatin to suppress fungal growth, and incubation proceeded at 28 °C for two weeks. Any colony showing a firm, leathery consistency, marked downward growth into the agar (substrate mycelium), and a dusty upper layer (aerial mycelium) was passaged onto International *Streptomyces* Project (ISP-2) media [10].

This isolation strategy was deliberately selective for actinomycetes rather than for the wider soil bacterial community. Starch casein agar supplemented with nystatin, combined with prolonged incubation at 28 ℃, favours the slow-growing, filamentous, substrate- and aerial-mycelium-forming colony morphology characteristic of actinomycetes while suppressing fast-growing Gram-negative bacteria and fungal contaminants; nystatin specifically inhibits fungal growth without affecting bacterial development [7]. This selective approach was adopted because actinomycetes, and *Streptomyces* in particular, are an established and prolific source of the extracellular reducing and capping biomolecules needed for green nanoparticle synthesis, as noted in the Background; the isolation protocol was therefore designed around this target group from the outset rather than as a general survey of soil bacterial diversity.

To obtain a pure, axenic isolate from the initial passage, presumptive actinomycete colonies were sub-cultured by repeated streaking onto fresh ISP-2 medium for eight successive rounds until each plate displayed a single, morphologically uniform colony type. Purity was confirmed at each round by consistent colony morphology (leathery texture, substrate and aerial mycelium formation) and by Gram staining showing a single, uniformly Gram-positive, branching filamentous morphotype with no contaminating unicellular bacterial or fungal forms; the confirmed pure isolate was then maintained on ISP-2 slants at 4 ℃ and as glycerol stocks at -80 ℃ for subsequent identification and biomass production.

### Identification of the actinomycete isolate

Morphology of the purified isolates was examined by Gram staining [11]. The purified isolate was then assigned an identity through 16 S rRNA gene sequencing. With the bacterial 16 S ribosomal RNA gene as the target, the recovered sequence was checked against NCBI records by means of BLAST. The top match returned 100% query coverage together with 100% sequence identity to *Streptomyces enissocaesilis*, while the phylogenetic tree that was generated placed it firmly among the high G + C Gram-positive *Streptomyces* lineage [12].

### Cultivation and preparation of actinomycete biomass

Under aerobic shaking at 30 °C and 150 rpm, the chosen actinobacterial strain was grown in starch nitrate broth for 96–120 h. Once incubation was complete, the biomass was recovered by centrifugation at 5000 rpm for 20 min and rinsed three times with sterile distilled water so that leftover medium constituents capable of driving non-specific chemical reduction were removed [13].

### Extracellular biosynthesis of AuNPs

After washing, the actinobacterial biomass was taken up again in sterile distilled water and held at 28 °C for a further 48 to 72 h. Passing this suspension through a 0.22 μm membrane then yielded a clear, cell-free filtrate. To trigger AuNP biosynthesis, an aqueous solution of chloroauric acid (HAuCl_4_.4H_2_O, crystalline powder, 99.9%, Oxford Lab Fine Chem LLP, Maharashtra, India) was introduced into the cell-free filtrate up to a final concentration of 1 mM. The mixture was next heated to boiling in a water bath over 30 min while being stirred at 150 rpm in the dark. The successful biosynthesis of AuNPs was visually confirmed by a color change that deepened from pale yellow to ruby red. AuNPs obtained from centrifugation at 10,000 rpm for 20 min were thoroughly rinsed with distilled water, lyophilized, and prepared as dry AuNP powders.

Sterile distilled water, rather than growth medium or buffered saline, was used for this resuspension step because it is free of the extraneous ions, chelators, and nutrient salts that can compete with Au³⁺ for reduction or interfere with subsequent nanoparticle nucleation, a precaution widely adopted in microbial green-synthesis protocols to keep the reaction system compositionally simple [10]. Resuspension in distilled water is hypo-osmotic relative to the growth medium and could plausibly alter extracellular metabolite secretion relative to standard growth conditions; this is an accepted trade-off in the green-synthesis literature rather than one specific to the present protocol. Comparing metabolite profiles and reduction efficiency under isotonic versus hypotonic resuspension conditions is a worthwhile direction for future optimisation of this system. Formal optimisation of the biosynthesis reaction itself (precursor concentration, temperature, and reaction time) was not performed in the present study, which used a single fixed set of conditions (1 mM HAuCl4, boiling water bath, 30 min); systematic optimisation of these parameters is noted as a direction for future work in the Conclusion.

### Physicochemical characterization of AuNPs

Morphology and particle-size distribution were assessed as previously reported [14] using transmission electron microscopy (TEM), elemental composition by EDX, crystallinity by X-ray diffraction (XRD), colloidal stability by zeta-potential analysis, and surface chemistry by FTIR spectroscopy. The employed instrumentation included a JASCO V-760 UV-Vis spectrophotometer, a JEOL TEM system, a Shimadzu LabX XRD-6000 diffractometer, a Malvern Nano-ZS90 zeta-potential analyzer, and a JASCO FT/IR-4100 instrument [15].

### Cell lines

Both MCF-7 human breast adenocarcinoma cells (ATCC^®^ HTB-22™) and normal peripheral blood mononuclear cells (PBMC) were grown in Dulbecco’s Modified Eagle Medium (DMEM) that contained 10% fetal bovine serum (FBS) together with 1% penicillin-streptomycin, the cultures being kept at 37 °C inside a humidified 5% CO_2_ incubator. Ahead of AuNP treatment, the cells were plated at 5 × 10^3^ cells/well in 96-well plates and left for 24 h [16].

### Cytotoxicity assay

A colorimetric metabolic assay built on the MTT method served to gauge cytotoxicity. Across a 48 h exposure, the prepared AuNPs were applied at 3.125, 6.25, 12.5, 25, 50, and 100 µg/mL to PBMC normal cells and to MCF7 breast cancer cells alike. Since the percent-inhibition values were referenced to a control absorbance of 0.700, that figure was used to cross-check the reported inhibition percentages. From the triplicate readings (three technical replicate wells within a single experiment), the mean absorbance, standard deviation (SD), standard error (SE), coefficient of variation (CV%), 95% confidence intervals, and dose-response fitting were derived [17].

### Annexin V-FITC/PI apoptosis assay

MCF-7 cells were treated with AuNPs at 100 µg/mL for 48 h prior to staining. To measure apoptosis, Annexin V-FITC/PI staining was paired with flow cytometry, an approach that separates viable cells (Annexin V-/PI-) from early apoptotic cells (Annexin V+/PI-), late apoptotic cells (Annexin V+/PI+), and necrotic or membrane-compromised cells (Annexin V-/PI+) [18].

### Cell-cycle analysis

Distribution across the cell cycle was established through PI-based DNA-content flow cytometry, a method that distinguishes the fractions of cells residing in G0/G1, S, and G2/M phases. Quantitative cell-cycle readouts were obtained for the untreated control, doxorubicin-treated, and AuNPs-treated (100 µg/mL, 48 h) groups [19–21].

### 5-bromo-2’-deoxyuridine (BrdU) proliferation assay

Proliferation of the cells was quantified with the BrdU Cell Proliferation ELISA Kit (Roche, Cat. No. 11647229001), used essentially as the manufacturer directs but with slight adjustments. In brief, MCF-7 cells received a range of AuNPs concentrations over 48 h. A BrdU labeling solution (10 µM final) was then introduced for a further 24 h so the label could be taken up into newly made DNA. The cells were fixed, the DNA denatured with HCl, and an anti-BrdU-POD antibody applied over 90 min at room temperature. Following the washes, TMB substrate was added and the absorbance read at 450 nm (reference 690 nm) on a microplate reader (BioTek Synergy 2). Inhibition of proliferation was computed as:

$$\:\%\:inhibition=[1-\left(\frac{Absorbance\:ofsample}{Absorbance\:of\:control}\right)X\:100$$

Data representing mean ± SD from three independent experiments (*n* = 6 wells/experiment). IC_50_ was determined by nonlinear regression using SPSS [21].

### Confocal laser scanning microscopy (CLSM)

MCF-7 cells were treated with AuNPs at 100 µg/mL for 48 h prior to AO/PI staining. To characterize how the cells died, an acridine orange/propidium iodide (AO/PI) double-staining solution was made by combining equal volumes of AO (100 µg/mL) and PI (100 µg/mL) in phosphate-buffered saline (PBS). Once treatment had finished, the medium was discarded and the cells given two rinses in cold PBS. The AO/PI mixture was then applied and left on the cells in the dark at room temperature for 10–15 min. A further gentle PBS wash cleared away surplus dye, after which the cells were taken straight to imaging [22]. Imaging of the stained cells proceeded on a confocal laser scanning microscope (e.g., Leica SP8 or Zeiss LSM), where:

- AO (Green): excitation was set at 488 nm, with emission recorded in the 500–530 nm range.
- PI (Red): excitation was set at 543 nm (or 561 nm), with emission recorded above 600 nm.

Images were obtained from various randomly selected fields. The dead-cell ratio was determined using image analysis software [23] to determine the ratio of PI-positive (dead) cells to the overall cellular population.

### Quantitative real-time PCR (RT-qPCR)

MCF-7 cells which were untreated as well as those treated with AuNPs at a concentration of 100 µg/mL for 48 h, had total RNA isolated using the TRIzol Reagent (Invitrogen, Cat. No. 15596026) with an additional chloroform extraction. RNA integrity was assessed with a NanoDrop 2000 spectrophotometer (A_260_/A_280_ = 2.0-2.1). The reverse transcription of RNA to complementary DNA (cDNA) was performed using the High-Capacity cDNA Reverse Transcription Kit (Applied Biosystems, Cat. No. 4368814) and was done according to the provided instructions.

On the QuantStudio 3 Real-Time PCR System (Applied Biosystems), RT-qPCR was performed with PowerUp SYBR Green Master Mix (Thermo Fisher, Cat. No. A25742). The housekeeping gene GAPDH was used for data normalization [24]. The primer sequences, included in Table S1, were designed using Primer-BLAST. The 2^^−ΔΔCt^ method was used to determine the relative expression of genes with the untreated cells serving as the calibrator (value assigned 1.00). The results were based on three independent experiments.

### Molecular docking

Molecular docking studies were utilized to predict possible interactions of biomolecule-associated gold complexes with molecular targets critical to breast cancer. The crystal structure of ERα (PDB ID: 3ERT) [25] and Cyclin-Dependent Kinase 2 (CDK2) (PDB ID: 1HCK) [26] were downloaded from the Protein Data Bank (PDB). The selection of ERα was due to its well-defined role in estrogen receptor positive breast cancer, particularly in MCF-7 cells [27, 28]. CDK2 was selected due to its role in the regulation of the cell cycle and the promotion of cancer cell proliferation [29].

Protein preparation was carried out using BIOVIA Discovery Studio Visualizer 2021 through removal of water molecules and co-crystallized ligands, followed by hydrogen addition and charge assignment. The prepared protein structures were subsequently converted to PDBQT format using AutoDock MGL Tools [19]. Binding sites were identified based on the coordinates of the co-crystallized ligands within the active sites of both proteins. Docking grid parameters were defined around the active-site regions, and the corresponding grid box coordinates and dimensions are provided in Table 1.

**Table 1:** Grid box coordinates and dimensions used for molecular docking simulations

Target protein	PDB ID	center_x (Å)	center_y (Å)	center_z (Å)	size_x (Å)	size_y (Å)	size_z (Å)
Estrogen receptor alpha (ERα)	3ERT	30.282	−1.913	24.206	20	22	20
Cyclin-dependent kinase 2 (CDK2)	1HCK	101.714	97.191	82.216	22	24	20

Serving as the test ligand, the cysteine–gold (Cys–Au) complex was employed as a simplified representative molecular motif of sulfur-containing biomolecules associated with the biological corona of the biosynthesized AuNPs, as suggested by the FTIR analysis [10, 12].

Marvin Sketch was used to build the ligand structures, which were then translated into docking-ready formats with Open Babel. The docking simulations themselves ran in AutoDock Vina, following the protocol earlier reported by Trott and Olson [13]. To keep the metal coordination geometry intact, the Cys-Au complex was handled as a rigid ligand. Binding affinities were reported as binding free energies (ΔG, kcal/mol).

The docking poses and their interactions, including hydrogen bonds and hydrophobic interactions, were analyzed using BIOVIA Discovery Studio Visualizer 2021. The interaction profiles were compared with those of the co-crystallized ligands using the Protein Plus web server [15]. This was done to assess the validity of the binding modes and their possible relationship to the anticancer activity against MCF-7 breast cancer cells.

### Statistics

All quantitative analyses were conducted in SPSS v 27. Absorbance values across the tested concentrations were compared by one-way ANOVA together with Tukey’s HSD post hoc test. Linear regression served to examine how log 10 concentration related to percent growth inhibition, and an approximate IC_50_ value was approximated through a constrained Hill model. A threshold of statistical significance was set at *p* < 0.05. For each assay, the number of independent biological replicates and, where applicable, the number of technical replicates per biological replicate are stated explicitly in the corresponding Results subsection; exact p-values, rather than inequality statements alone, are reported throughout the Results wherever the exact value was available from the statistical output.

## Results

### Identification of the actinomycete isolate

**Fig. 1:**
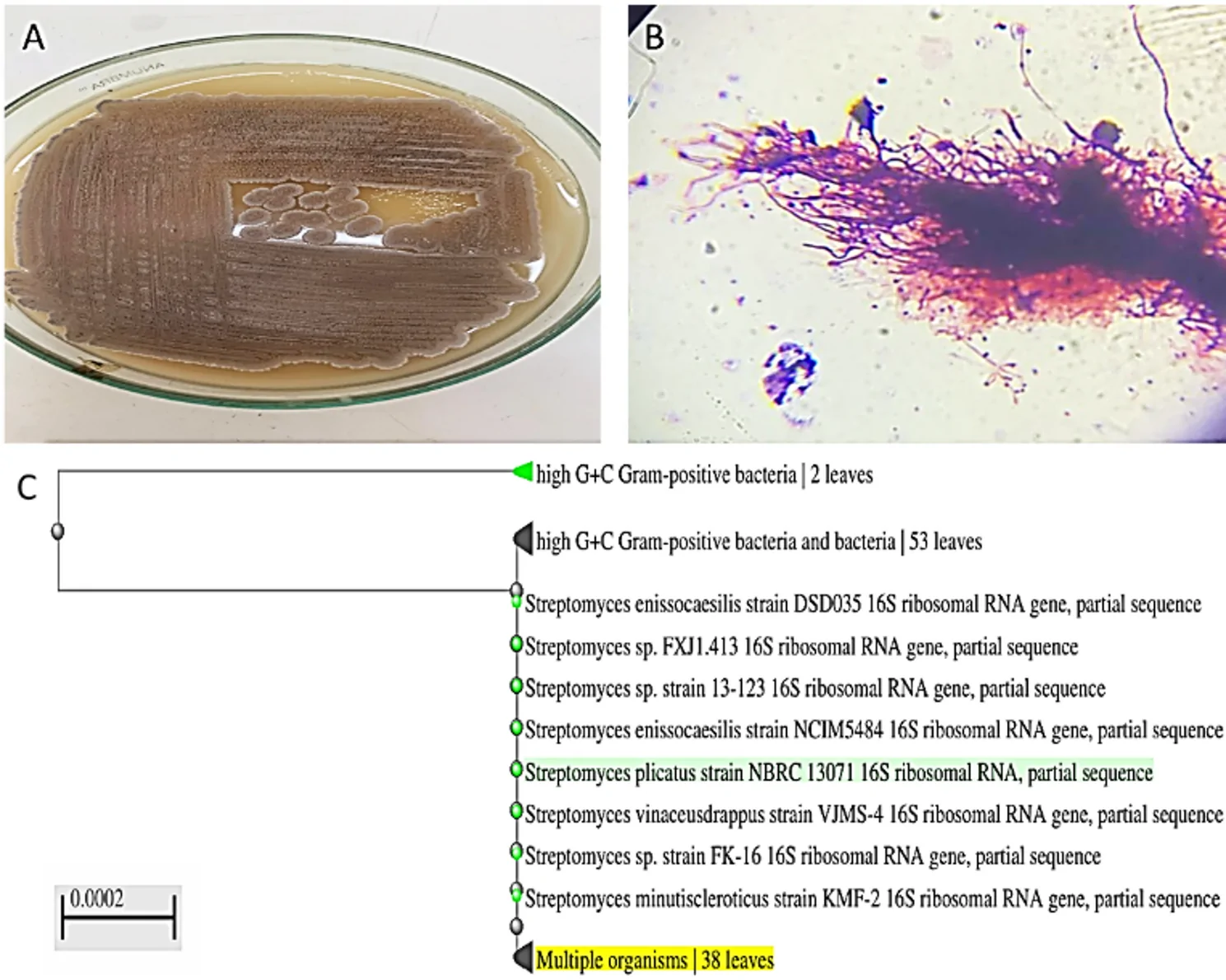
Morphological identification of the isolated actinomycete from soil **A** on starch casein agar and **B** under microscope after Gram stain. **C** Phylogenetic tree of the actinomycete isolate based on the 16 S rRNA sequence

On SCA plates, the isolates presented a marked brown pigmentation to the naked eye, forming dark-brown, leathery colonies topped by a dense aerial mycelium (Fig. 1A). Under the microscope, Gram staining showed the isolates to be Gram-positive, taking on a violet hue (Fig. 1B). Their clearest microscopic trait was a network of slender, heavily branching filamentous hyphae (mycelia). The 16 S rRNA sequencing report pinpointed the isolated actinomycete as *Streptomyces enissocaesilis*, returning 100% query coverage and 100% sequence identity against accession MW217140.1 (Table 2).

**Table 2:** Molecular identification of the actinomycete isolate

Feature	Result	Feature	Result
Target gene	16 S rRNA gene	Read length	661 bp
Best BLAST hit	*Streptomyces enissocaesilis*	Accession	MW217140.1
Query cover	100%	Identity	100%
Taxonomic position	Actinomycetota / Streptomycetales	Interpretation	Actinobacterial isolate suitable for green synthesis

On the phylogenetic tree, the isolate fell inside the high G + C Gram-positive bacterial branch, sitting in close affinity with *Streptomyces* taxa (Fig. 1C).

The phylogenetic tree shown in Fig. 1C was generated using the NCBI BLAST Tree View tool based on the 16 S rRNA gene sequence and was constructed using the Neighbor-Joining (NJ) method based on sequence distances; this tool does not provide bootstrap analysis, so bootstrap support values are not reported here, instead assigning unsupported values. The isolate clustered most closely with recognized *Streptomyces* reference sequences, including sequences assigned to *Streptomyces enissocaesilis* and other closely related taxa, to the exclusion of other actinomycete genera. This close, well-supported grouping, combined with the 100% BLAST identity to *Streptomyces enissocaesilis* (Table 2), provides convergent, independent support for the species-level identification used throughout this study. The 661 bp 16 S rRNA read length reported in Table 2 is sufficient to support a confident genus- and species-level BLAST match at 100% identity and coverage, though it is shorter than the near-full-length (about 1400 to 1500 bp) sequence now considered best practice for definitive taxonomic and phylogenetic placement of a novel actinomycete isolate.

### AuNPs characterization

**Fig. 2:**
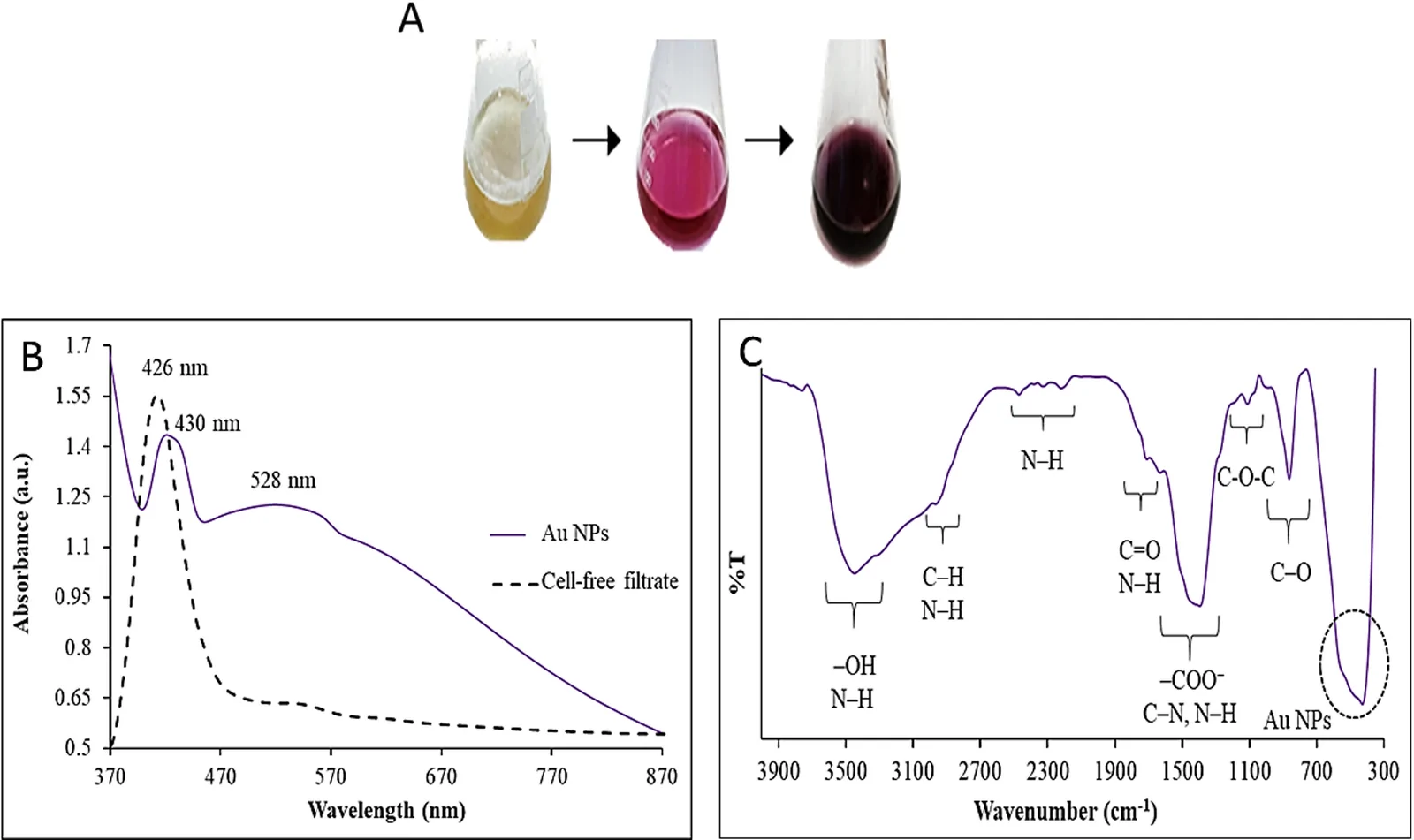
**A** The color transition from the pale yellow of the HAuCl_4_ precursor to ruby red and eventually a deep purple of AuNPs. **B** UV-Vis spectrum of the biogenic AuNPs. **C** FTIR transmission spectrum of biosynthesized Au NPs

Effective bio-reduction of gold ions to AuNPs by *Streptomyces enissocaesilis* was tracked through the stepwise colour change away from the pale yellow of the HAuCl_4_ precursor, moving to ruby red and ultimately a deep purple over 48 h (Fig. 2A). For the biosynthesized AuNPs, the UV-Vis absorption spectra presented an distinct optical profile carrying two distinct peaks. Based on established convention in the AuNP literature, a primary SPR maximum in the 520 to 530 nm range is generally taken as consistent with spherical or quasi-spherical particles with diameters of roughly 20 to 50 nm [1], and the 528 nm maximum recorded here for the present biosynthesized AuNPs, discussed further below in relation to the TEM sizing data, is in line with that convention rather than being itself a novel size measurement. A second sharp peak, notably, appeared near 430 nm. In actinobacterial biosynthesis such a dual-peak pattern usually reflects one of two situations: here the 430 nm band corresponded to abundant secretion of actinobacterial metabolites, among them flavins or phenolic compounds, that operate as the chief reducing and capping agents. Reduction in the present case is most likely driven by the actinomycete’s extracellular secondary metabolites together with its NADH-dependent nitrate reductases, which are probably the agents behind the efficient reduction recorded in this work. Strong support for the participation of proteinaceous or pigment-based capping agents, which stabilize the AuNPs and guard against irreversible agglomeration, comes from the 430 nm peak (Fig. 2B).

A shift toward purple after prolonged incubation is commonly associated with nanoparticle aggregation in biogenic AuNP systems. All physicochemical characterization (UV-Vis, TEM, zeta potential, XRD, EDX, FTIR) and all biological testing reported in this manuscript were performed on the 48 h ruby-red preparation, harvested and lyophilized at that time point rather than at 72 h. The reference to a deep-purple transition at 72 h describes a preliminary observation made on preparations left longer than the 48 h point actually used, retained here only to document that this system shows time-dependent colour change on extended incubation, as other biogenic AuNP preparations do.

A rich set of absorption bands in the FTIR spectrum points to a stable, multi-component biological corona arising from the actinomycete extracellular matrix (Fig. 2C). A broad, prominent absorption band centred near 3440 cm^− 1^ arises from stretching of hydroxyl (-OH) groups in polysaccharides or phenolic compounds and from N-H stretching of primary amines within proteins. The surface of these nanomaterials indicates significant hydrogen bonding, which is conducive to maintaining the stability of the dispersed colloidal system within water. A strong peak at 1635 cm^− 1^ indicates the stretching of the carbonyl (C = O) in the peptide bonds of the proteins and clearly defines the amide I band. This observation indicates that the Nanoparticle (NP) surface interacts with the secreted proteins and/or enzymes. The feature at 1405 cm^− 1^ is either stretching of the carboxylate group (-COO^−^) and/or C-N stretching and N-H bending (amide II). The amide I and II bands suggest that the main stabilizers are proteins of actinomycete origin. These proteins, which form a biological corona through steric hindrance and prevent NP aggregation, anchor to the AuNPs via cysteine residues or free amine groups. The groups that are identified here, carboxyl and hydroxyl groups, which behave as electron donors, very likely assist in the reduction of Au³⁺ to Au⁰. A strong peak near 1075 cm^− 1^ is indicative of C-O-C stretching, which corresponds to bioactive polysaccharides’ skeletal ether linkages. The defined peaks below 700 cm^− 1^, sitting at lower wavenumbers, are representative of the collective vibrations of Au-N or Au-O bonds and point to direct coordination of the organic ligands to the metal core. The dense organic corona prevents the NP core from oxidation and coalescence as reported in green chemistry. These FTIR findings, when combined with the UV-Vis spectra, indicate temporal stability.

The band assignments given above (hydroxyl/amine stretching near 3440 cm^− 1^, amide I near 1635 cm^− 1^, amide II/carboxylate near 1405 cm^− 1^, C-O-C polysaccharide stretching near 1075 cm^− 1^, and Au-N/Au-O coordination bands below 700 cm^− 1^) follow standard FTIR assignment conventions for protein- and polysaccharide-capped biogenic metal nanoparticles. These assignments would be considerably strengthened by a direct point of comparison: the FTIR spectrum of the cell-free actinomycete filtrate used as the reducing/capping agent, run under the same conditions as the AuNP spectrum, was not included in the present figures. This filtrate spectrum is being acquired for inclusion as an additional panel in Fig. 2, which will allow the peaks retained, shifted, or lost upon nanoparticle formation to be identified directly rather than inferred from literature precedent alone, strengthening the evidence that the assigned biomolecules are genuinely involved in AuNP reduction and capping in this specific system.

**Fig. 3:**
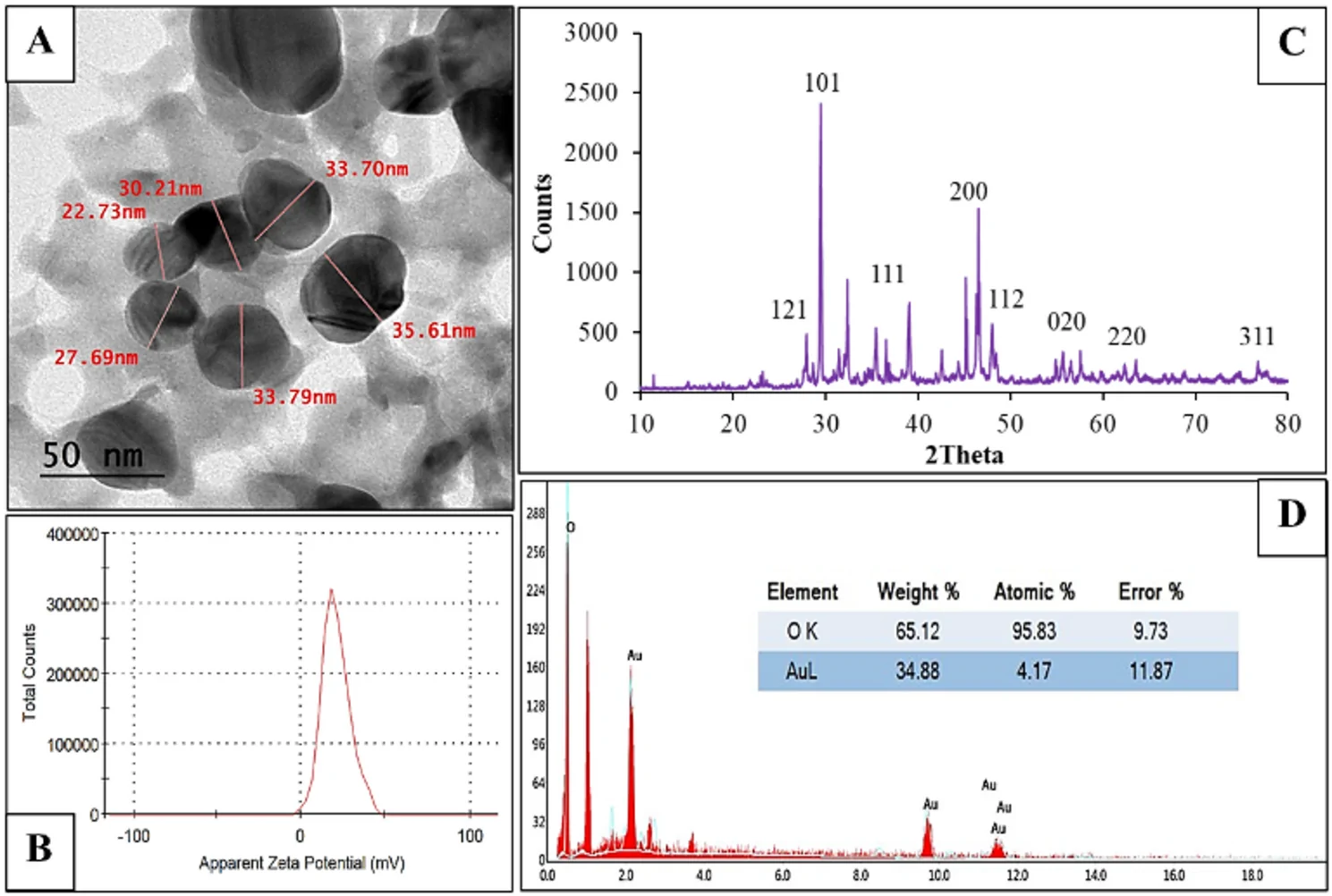
Comprehensive characterization of actinomycetes-mediated AuNPs. **A** TEM micrograph showing particle morphology and size, **B** zeta potential distribution curve, **C** the XRD diffractogram, and **D** the EDX spectrum illustrating elemental composition and high oxygen content from the bio-corona

The size distribution and shape of the biosynthesized AuNPs were clarified by TEM examination (Fig. 3A). In shape, the AuNPs were mostly ellipsoidal and quasi-spherical. The strong biological capping evidenced in the FTIR data explains their even spreading, the lack of obvious irreversible aggregation, and the absence of irreversible aggregation. Quantitative assessment of the TEM micrographs identified the sizes of the AuNPs to be in the range of approximately 22.73 to 35.61 nm. The zeta potential measurements (Fig. 3B) examined the surface charge and long-term stability of the AuNPs, given their size and time of preparation. The distribution curve showed one sharp peak positioned at + 27 mV. For biogenic synthesis, + 27 mV reflects moderate-to-good stability afforded by electrosteric stabilization, even though the threshold conventionally taken for excellent colloidal stability is a zeta potential exceeding 30 mV in absolute value [30]. This clustering of a positive charge suggests that positively charged actinomycete proteins cap the NPs. The crystalline structure and phase purity of the biosynthesized AuNPs were confirmed by XRD (Fig. 3C). A number of sharp diffraction peaks were recorded as evidence for the presence of a face-centered cubic lattice of metallic Au. The principal Bragg reflections were assigned at 38.1° (111), 44.4° (200), 64.5° (220), 77.6° (311) for 2θ values and corresponded to JCPDS No. 04-0784. Notably, the pattern additionally carried several weaker peaks at 101, 112, and 020. Although the AuNPs were washed multiple times with distilled water after synthesis, which would be expected to remove freely dissolved salts, these minor peaks more plausibly reflect crystallization of components of the tightly bound bio-organic corona itself, for example proteinaceous or polysaccharide material that co-precipitates with the metallic core and is not removed by aqueous washing, rather than residual unbound salt from the reaction filtrate. The elemental composition was established through EDX analysis (Fig. 3D). Strong Au signals in the spectrum at 2.1 keV (Lα) and near 9.7 keV (Lβ) confirm that chloroauric acid was significantly reduced. The sample comprised roughly 34.88% Au and 65.12% O. The dense bio-organic capping layer of proteins and carbohydrates wrapping the metallic core explains why O dominates the atomic count (95.83%). These O peaks lend further weight to the FTIR evidence that hydroxyl and carboxyl groups serve as stabilizing ligands. The physical characterization data for the biogenically synthesized AuNPs are summarized in Table S2.

### Cytotoxicity of the biogenic AuNPs

**Fig. 4:**
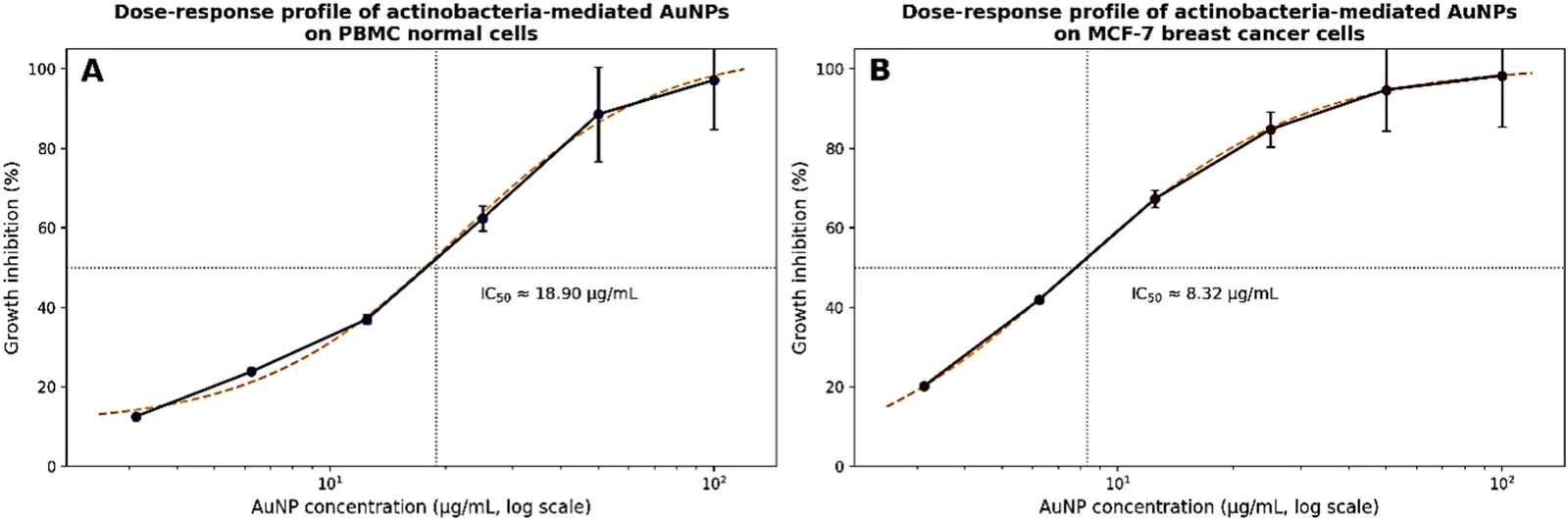
**A** The in vitro safety profile of biogenically synthesized AuNPs on PBMC normal cells, revealing the half-maximal inhibitory concentration (IC_50_) equal to 18.90 µg/mL. **B** The IC_50_ of the biosynthesized AuNPs on MCF7 cancer cells equal to 8.32 µg/mL

To probe how biocompatible the biosynthesized AuNPs are, their cytotoxic action on PBMC normal cells was examined over a concentration gradient running from 3.125 to 100 µg/mL. A concentration-dependent decline in the metabolic activity of the PBMC followed treatment with AuNPs. Mean absorbance dropped from 0.6125 at 3.125 µg/mL to 0.0196 at 100 µg/mL, equivalent to growth-inhibition values of 12.50% and 97.20%, respectively. Inhibition rose steadily across the mid-range of concentrations tested, climbing from 37.00% at 12.5 µg/mL to 62.39% at 25 µg/mL, before the curve flattened progressively toward saturation (Table S3 and Fig. 4A). Such a profile shows that the biosynthesized AuNPs were not merely cytostatic; instead they became strongly suppressive as concentration rose, reaching near-complete inhibition at 50–100 µg/mL.

A one-way ANOVA pointed to a highly significant difference in absorbance across the tested concentrations (F = 1244.7, *p* < 0.0001). According to Tukey’s HSD, every pairwise comparison between concentrations was significant at the 0.05 level, reflecting the low within-group variability at each dose. Linear regression likewise revealed a strong positive link between log10 concentration and inhibition percentage (*r* = 0.988, *p* = 0.00021) (Table S4).

### In vitro anticancer activity of AuNPs against MCF-7 Cells

The effect of the synthesized AuNPs on the human breast cancer line MCF-7 was studied from 3.125 to 100 µg/mL using the same MTT protocol applied to the PBMC assay. The gold nanoparticles acted on the MCF-7 cancer cells in a concentration-dependent manner, with the strongest inhibition observed at the highest concentration tested.

Mean absorbance dropped from 0.5590 at 3.125 µg/mL to 0.0117 at 100 µg/mL, equivalent to growth-inhibition values of 20.14% and 98.33%, respectively. The steepest rise in activity fell between 3.125 and 12.5 µg/mL, where inhibition climbed from 20.14% to 67.33%, after which the curve flattened progressively toward saturation (Table S5 and Fig. 4B). The half-maximal inhibitory concentration (IC_50_) of AuNPs against MCF-7 cells was determined to be 8.32 µg/mL (Fig. 4B). A one-way ANOVA confirmed a highly significant difference in absorbance across the tested concentrations (F = 3943.2, *p* < 0.0001), and linear regression showed a strong positive relationship between log10 concentration and inhibition percentage (*r* = 0.969, *p* = 0.00142) (Table S6). Comparing this IC50 with the PBMC value obtained under the same protocol (Sect.  3.3) gives a selectivity index (IC50 PBMC / IC50 MCF-7) of 2.27, indicating that, on this dose-response measure, the biosynthesized AuNPs inhibited MCF-7 cells at a considerably lower concentration than they inhibited normal PBMC cells, consistent with preferential potency against the cancer cell line rather than indiscriminate cytotoxicity.

**Fig. 5:**
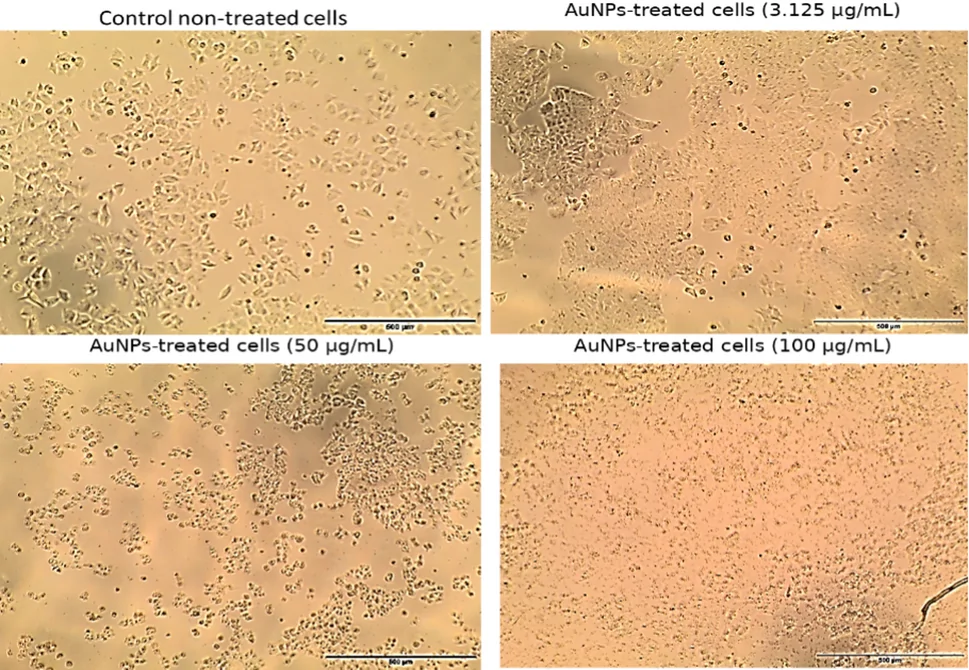
General cytotoxic morphological change in MCF-7 cells following AuNP treatment, consistent with reduced cell density and loss of the confluent, spread morphology seen in untreated cultures

At the magnification used for this micrograph, the AuNPs-treated cells showed an overall reduction in cell density and loss of the normal spread, confluent morphology of untreated cells, consistent with general cytotoxicity and cell loss rather than allowing the specific hallmarks of shrinkage or membrane disruption to be resolved at this resolution; the finer apoptotic morphology described in the following sections was instead assessed directly by confocal microscopy at higher magnification, as shown in Fig. 5.

### AuNP-induced apoptosis

**Fig. 6:**
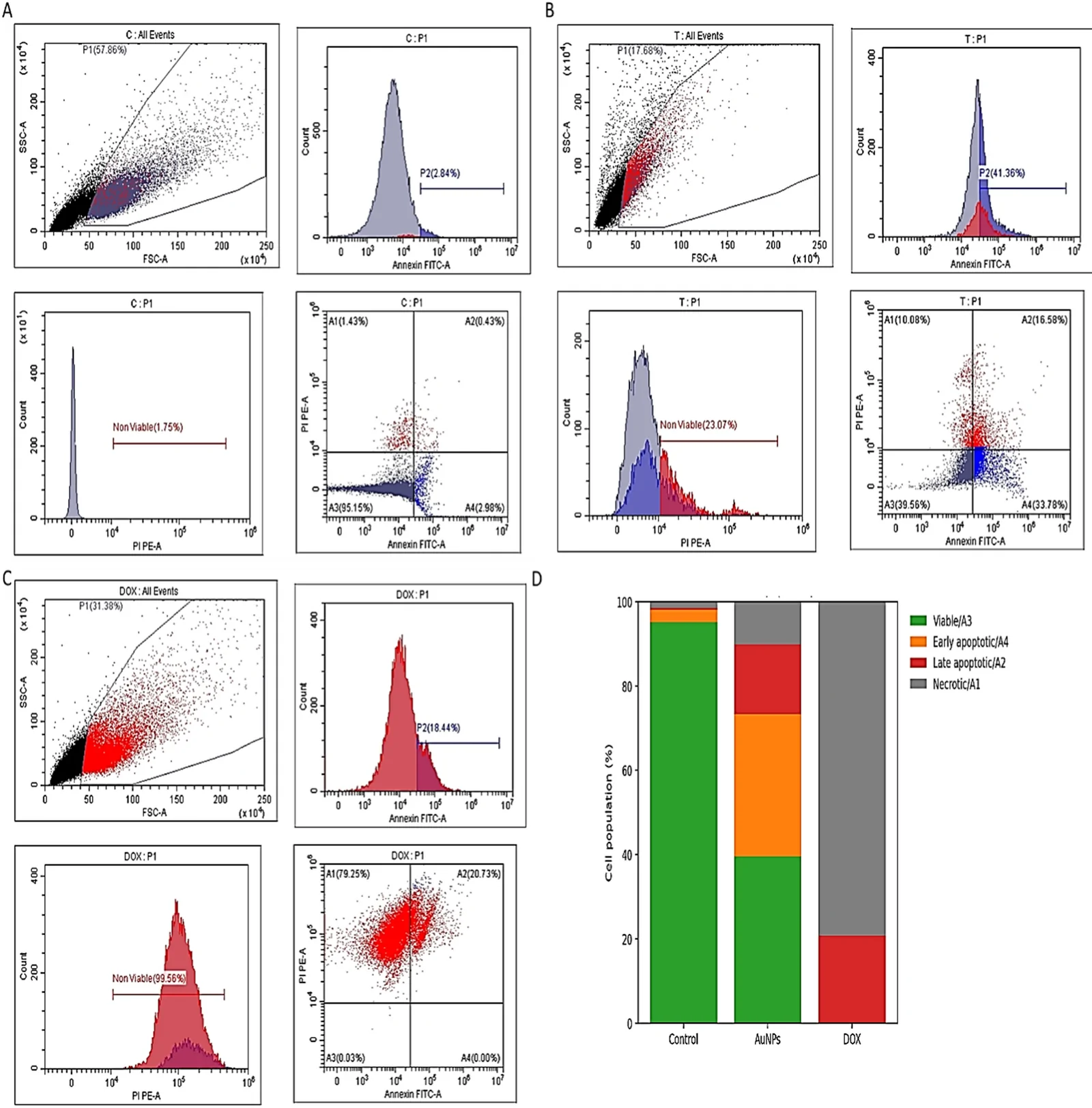
Annexin V-FITC/PI flow cytometric analysis of apoptosis in MCF-7 cells: **A** untreated control, **B** AuNPs-treated cells, **C** doxorubicin-treated cell, and **D** bar chart showing the distribution of viable, early apoptotic, late apoptotic, and necrotic/non-viable cell populations

As shown in Fig. 6, in the Annexin V-FITC/PI analysis, untreated control cells were mostly viable. 95.15% of the control cells were located in Quadrant A3, and 2.98% of the cells were in the early apoptotic phase. 0.43% of the cells were in the late apoptotic phase, and 1.43% of the cells were necrotic and non-viable. Treatment of cells with AuNPs resulted in a significant decrease in cell viability, with 39.56% of the cells remaining viable. Increased percentages of cells were in the early apoptotic phase (33.78%), late apoptotic phase (16.58%), and necrotic non-viable cells (10.08%). The total percentage of cells in the early and the late apoptotic phase, combined, was 50.36%, indicating that the predominant response of cells treated with AuNPs was to undergo apoptosis. A more severe and irreversible response was observed with treatment of cells with doxorubicin. Virtually no cells were viable after doxorubicin treatment, and 79.25% of the treated cells were necrotic and non-viable.

### Cell-cycle analysis

**Fig. 7:**
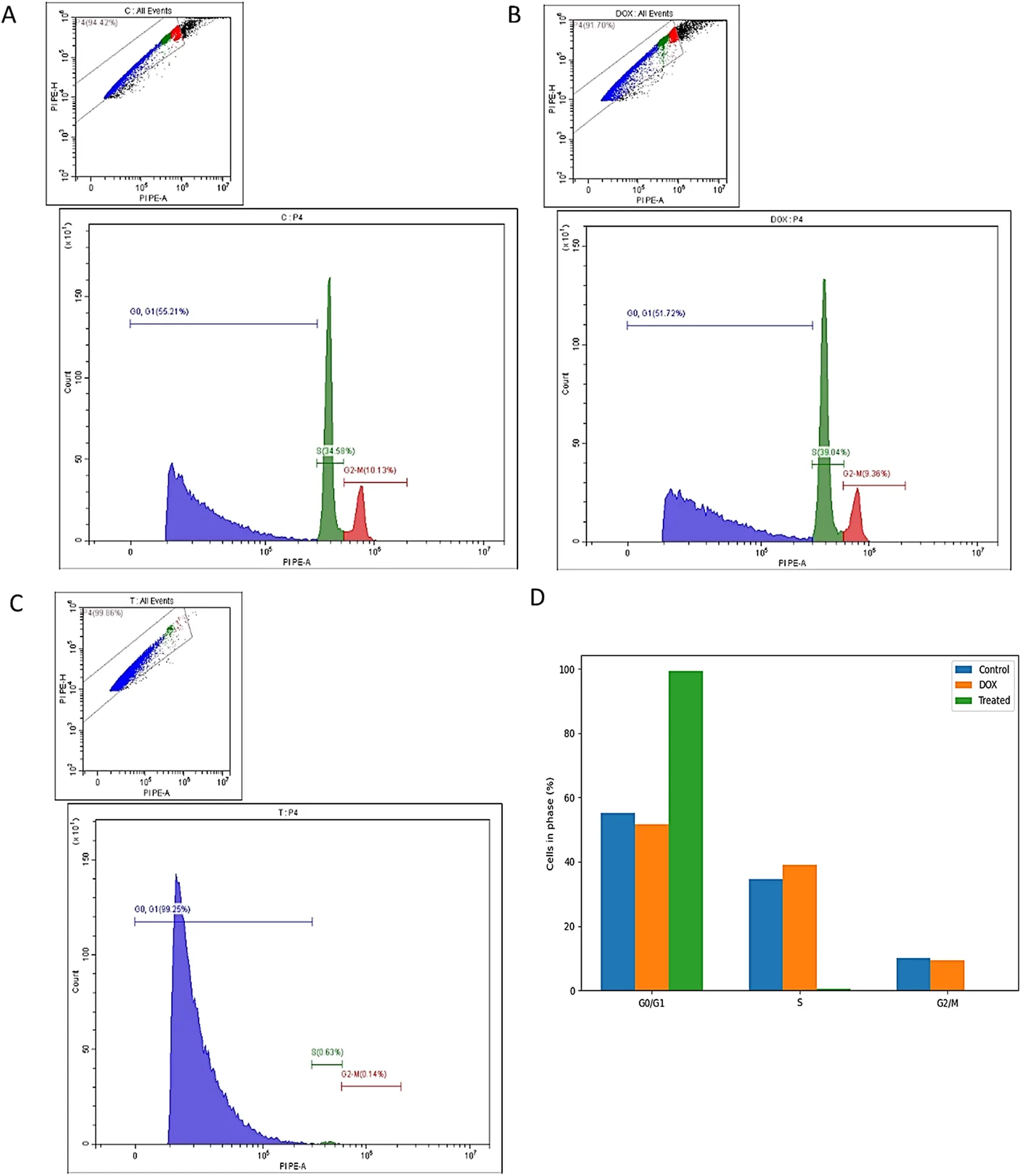
Cell cycle flow cytometry outputs for: **A** the control non-treated, **B** doxorubicin-treated, and **C)** AuNPs-treated cells. **D** Bar graph showing cell cycle distribution in different cells

The three groups’ cell cycle profiles show distinct distributions. The untreated control cells showed a normal distribution, with most cells in G0/G1 and many cells in S and G2/M phases. The control group’s distribution showed an active distribution of DNA synthesis and mitosis. The doxorubicin-treated group exhibited a distribution consistent with the cytotoxic and cell cycle disruptive properties of doxorubicin. Doxorubicin is known to disrupt cell cycle dynamics and inhibit DNA synthesis. The most drastic distribution change was observed with AuNPs. With 99.25% of cells in G0/G1, and 0.63% in S, and 0.14% in G2/M (Table S7 and Fig. 7), it seems that AuNP treatment strongly inhibited and practically blocked the cells from entering and progressing through the DNA synthesis and mitosis. A marked accumulation in G0/G1 indicates that AuNPs enforced a strict cell cycle arrest, resulting in a complete halt in cell proliferation.

### BrdU assay

Augmented effects of gold nanoparticles (AuNPs) resulted in an increased inhibition of DNA synthesis (BrdU incorporation) as a function of an increasing AuNP concentration. The 100 µg/mL AuNP concentration caused a 78.4 ± 3.2% inhibition (strong suppression of DNA synthesis/proliferation) compared to untreated controls (*p* < 0.001). The 25 and 50 µg/mL AuNPs produced a moderate inhibition of 32.1% and 56.7%, respectively. The 200 µg/mL concentration exhibited an almost total inhibition (92.6%). Read together with the cell-cycle data (Sect.  3.6), this concentration-dependent fall in BrdU incorporation is consistent with a G0/G1 cell-cycle arrest and a parallel increase in apoptosis of breast cancer cells, rather than a discrete S-phase block (see Discussion).

**Fig. 8:**
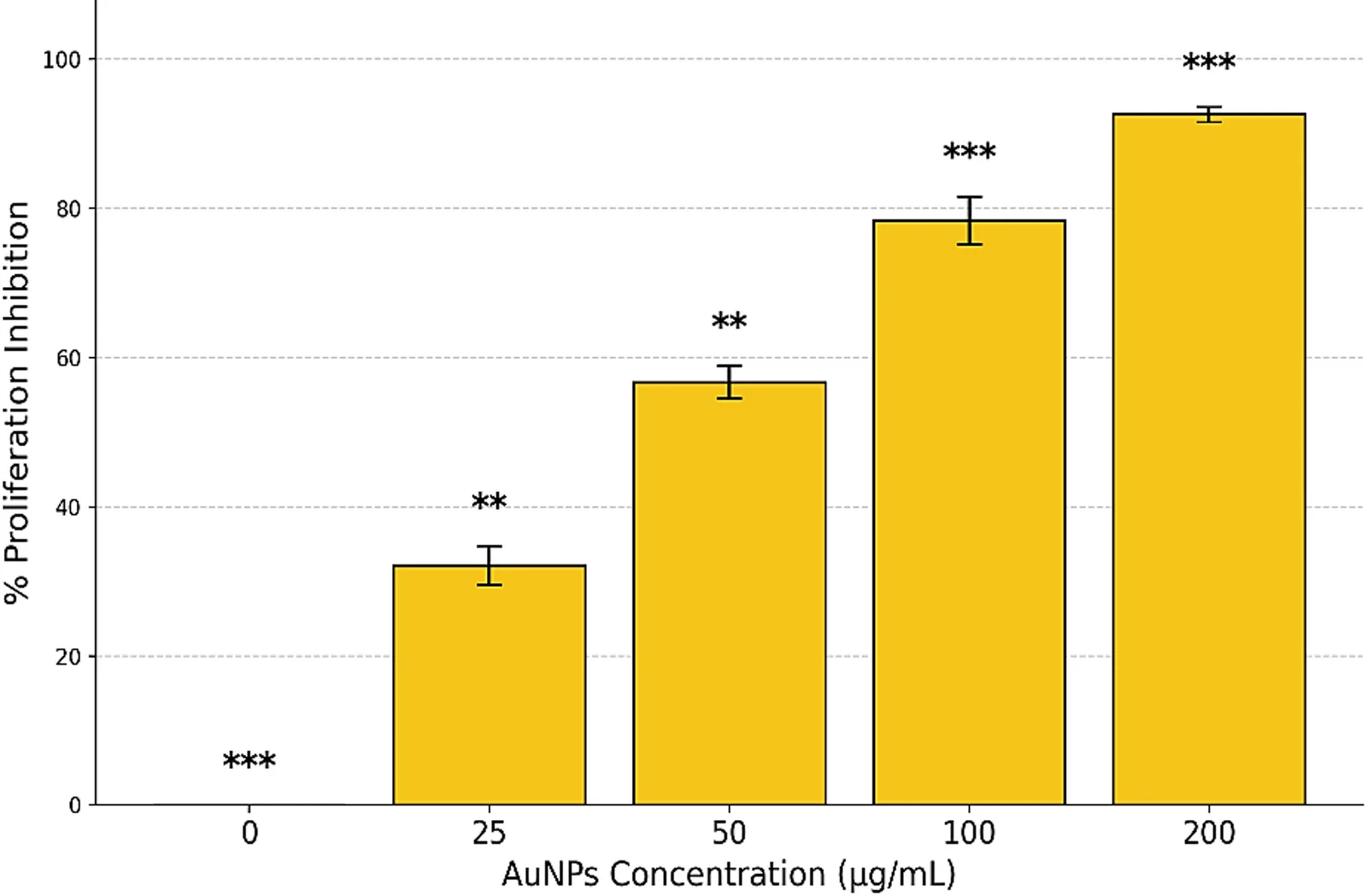
Dose-dependent inhibition of BrdU incorporation in MCF-7 breast cancer cells treated with biogenic AuNPs for 48 h

Concentration-dependent suppression of BrdU incorporation in MCF-7 breast cancer cells was recorded after 48 h of exposure to biogenic gold nanoparticles (AuNPs; 0–200 µg/mL) and a subsequent 24 h BrdU pulse-labeling. Values are reported as mean ± SD (*n* = 3 independent experiments, 6 replicates/condition). The AuNPs produced strong, dose-related inhibition of DNA synthesis (IC_50_ ≈ 62 µg/mL; r^2^ = 0.98, nonlinear regression; Table S8), reaching 78.4 ± 3.2% inhibition at 100 µg/mL (F_5_,_90_ = 247.3, *p* < 0.0001; one-way ANOVA). A Tukey’s post-hoc test verified significance for every concentration against the control (q > 8.2, *p* < 0.001), while 100 and 200 µg/mL did not differ significantly (q = 2.1, *p* = 0.45), pointing to a plateau consistent with the G0/G1-arrest-driven suppression of DNA synthesis described above, rather than a distinct S-phase blockade (Fig. 8).

Combined with the earlier decline in cell viability, a decrease in BrdU incorporation, and an increase in the apoptotic fraction, these data further support the hypothesis that AuNPs triggered an early cell cycle arrest and an apoptotic cell death, demonstrating a strong antiproliferative effect.

### CLSM of AuNPs-treated cells

**Fig. 9:**
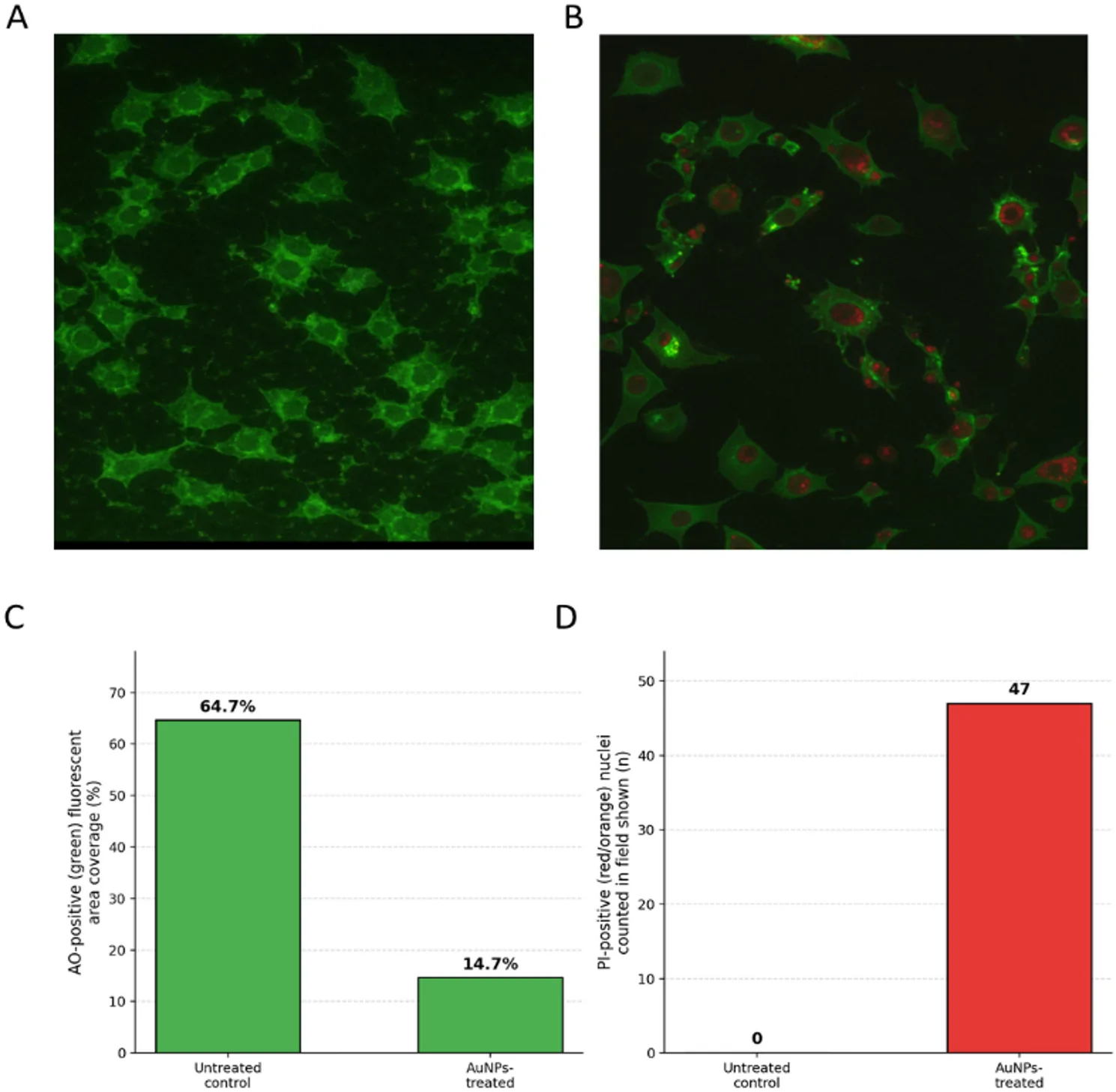
CLSM images of **A** untreated and **B** AuNPs-treated MCF-7 cells following AO/PI staining. Images are shown at the acquisition magnification used for this assay, sufficient to score nuclear AO/PI colour and density but not to resolve fine membrane-level structural detail. **C** Green (AO-positive) fluorescent area as a percentage of total field area. **D** Number of discrete PI-positive (red/orange) nuclei identified by watershed segmentation

Through the AO/PI dual-staining method, confocal microscopy captures the cytotoxic effect of AuNPs on MCF7 breast cancer cells, distinguishing the stages of cell death from membrane integrity and nuclear morphologyUnder the control condition (Fig. 9A) the cells exhibited bright and even green fluorescence which is indicative of high viability. As the nuclei of the cells remained intact, AO intercalated neatly into the double-strand DNA of the healthy cells. Red fluorescence (PI) was absent indicating that the membranes were intact and remained impermeable, which is expected for healthy and proliferative cell population.

After exposure to the AuNPs (Fig. 9B), cellular health shifted markedly, with visual assessment of representative fields indicating that approximately 65% of cells were apoptotic or dead. A sizeable share of cells took on red or orange-red nuclei, indicating that AuNP treatment had compromised plasma membrane integrity, letting PI enter and bind DNA so that it overrode the AO signal. Features consistent with apoptosis, including nuclear condensation and reduced cell density, were visible in the treated cells; because AO/PI staining alone cannot reliably distinguish early apoptosis, late apoptosis, and necrosis from one another, these observations are treated here as qualitative, corroborating evidence rather than as a precise quantitative or mechanistic classification, directionally consistent with the Annexin V-FITC/PI flow-cytometry data reported above. Yellow/orange cells are consistent with a transition from early toward late apoptosis as membrane damage grows, whereas bright red cells are consistent with necrosis or late-stage apoptosis. The overall cell density dropped against the control, consistent with the antiproliferative activity of the biosynthesized AuNPs. It should be noted that, at the magnification used for these images, fine structural hallmarks such as membrane blebbing or precise morphological shrinkage cannot be reliably resolved; the interpretation above therefore rests on the AO/PI fluorescence signal itself (nuclear colour and condensation) rather than on directly visualized membrane or shape changes, and higher-magnification imaging would be needed to substantiate structural claims of that kind.

As a supplementary, exploratory quantification of the two representative fields shown in Fig. 9A and B, we additionally performed an automated colour-threshold image analysis (Python/OpenCV): green (AO-positive) signal was quantified as the percentage of field area above a green-over-red intensity threshold, since the confluent, touching cell morphology in the untreated field makes discrete single-cell segmentation unreliable by simple thresholding; red/orange (PI-positive) nuclei were instead counted as discrete objects using distance-transform watershed segmentation on the red channel, which is better suited to separating touching nuclei (Fig. 9C and D). This automated analysis is a single-field, non-independently-validated estimate, not a substitute for a properly replicated, blinded manual count, and is reported here only as supplementary, exploratory evidence alongside the qualitative assessment above.

### Effect of AuNPs on c-erbB-2 and p53 expression using RT-qPCR

**Fig. 10:**
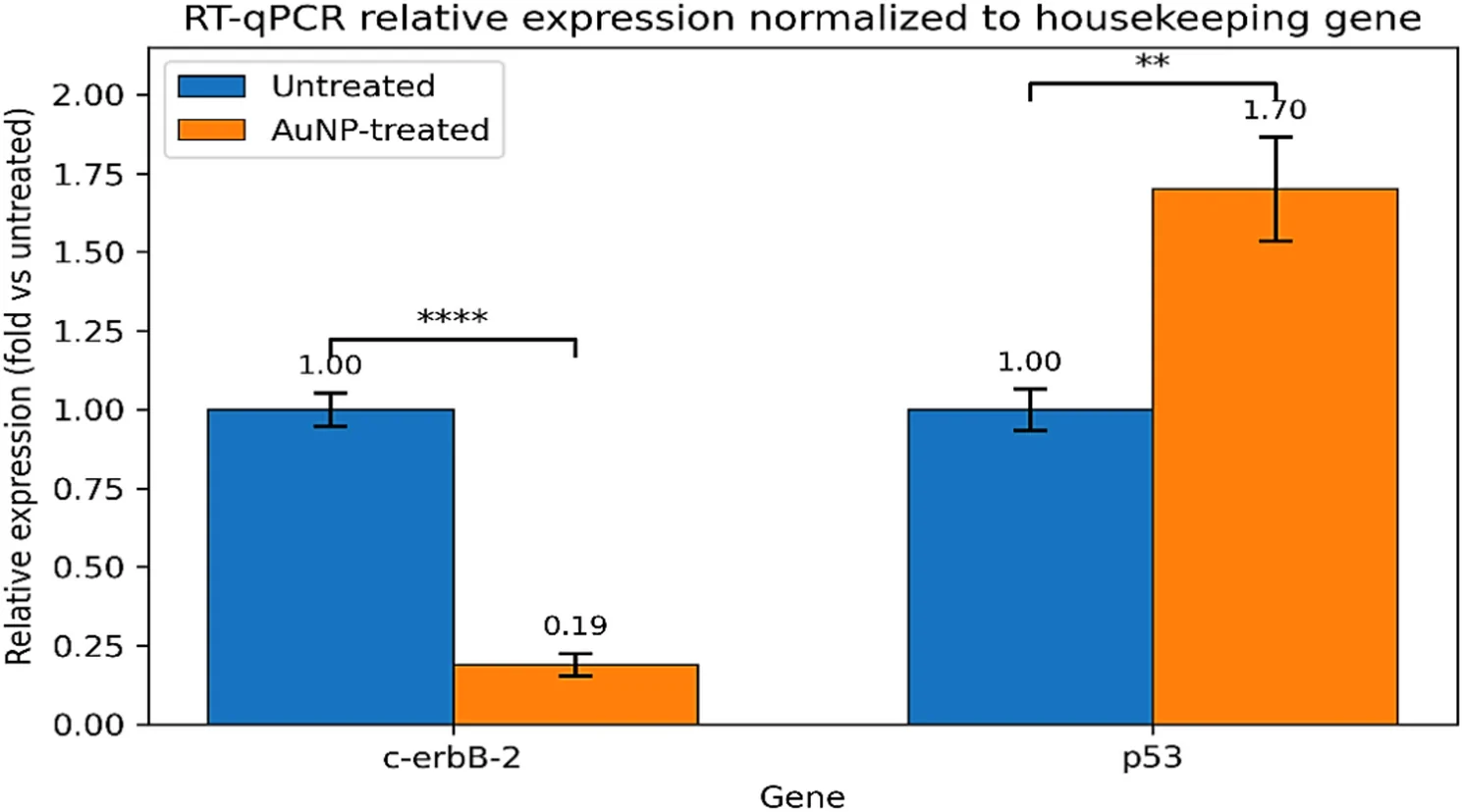
Relative expression of **c-erbB-2** and **p53** genes in the untreated and AuNPs-treated cells, as determined by RT-qPCR after normalization to the housekeeping gene. AuNPs treatment reduced **c-erbB-2** expression from **1.00** in untreated cells to **0.19-fold**, while **p53** expression increased from **1.00** to **1.70-fold**. Data are expressed as mean ± SD from three independent biological replicates (*n* = 3). Asterisks denote statistical significance versus the untreated calibrator by unpaired two-tailed Welch’s t-test (****p < 0.0001 for c-erbB-2; **p = 0.0099 for p53)

After normalization to the housekeeping gene, RT-qPCR revealed that the biosynthesized AuNPs substantially changed c-erbB-2 and p53 expression compared with untreated cells. Serving as the calibrator, the untreated cells were assigned a relative expression of 1.00 for each gene. In the AuNPs-treated cells, c-erbB-2 fell to 0.19-fold, an 81% reduction versus untreated cells, whereas p53 rose to 1.70-fold, a 70% increase over untreated cells (Table S9 and Fig. 10). Across three independent biological replicates, mean fold changes were 1.00 ± 0.05 (untreated) versus 0.19 ± 0.04 (AuNPs-treated) for c-erbB-2, and 1.00 ± 0.07 (untreated) versus 1.70 ± 0.17 (AuNPs-treated) for p53 (mean ± SD). Both differences were statistically significant by unpaired two-tailed Welch’s t-test (c-erbB-2: t = 21.91, *p* < 0.0001; p53: t = 6.82, *p* = 0.0099). Taken together, these findings show that AuNP treatment simultaneously reduced the oncogene c-erbB-2 and increased the tumor suppressor p53, shifting the molecular profile toward an antitumor state (Table 3).

**Table 3:** Summary of RT-qPCR relative expression results for c-erbB-2 and p53

Gene	Functional role	Untreated (mean ± SD)	AuNP-treated (mean ± SD)	Change vs. untreated	Statistics
c-erbB-2	Oncogene	1.00 ± 0.05	0.19 ± 0.04	−81%	t = 21.91, *p* < 0.0001
p53	Tumor suppressor	1.00 ± 0.07	1.70 ± 0.17	+ 70%	t = 6.82, *p* = 0.0099

### In silico results

To explore how the biomolecule-associated gold complexes might engage breast cancer-related targets, molecular docking was carried out against estrogen receptor alpha (ERα; PDB ID: 3ERT) [25] together with cyclin-dependent kinase 2 (CDK2; PDB ID: 1HCK) [26]. The ligand chosen was the cysteine-gold (Cys-Au) complex, standing in for the sulfur- and amide-linked biomolecular interactions present in the biological corona of the biosynthesized AuNPs.

Rather than relying on docking-score ranking alone, the docking poses were picked for their biologically meaningful interaction profiles and their fit within the active site. The computed binding affinities spanned − 3.1 to − 6.1 kcal/mol, signifying moderate yet favourable interactions with both target proteins.

**Fig. 11:**
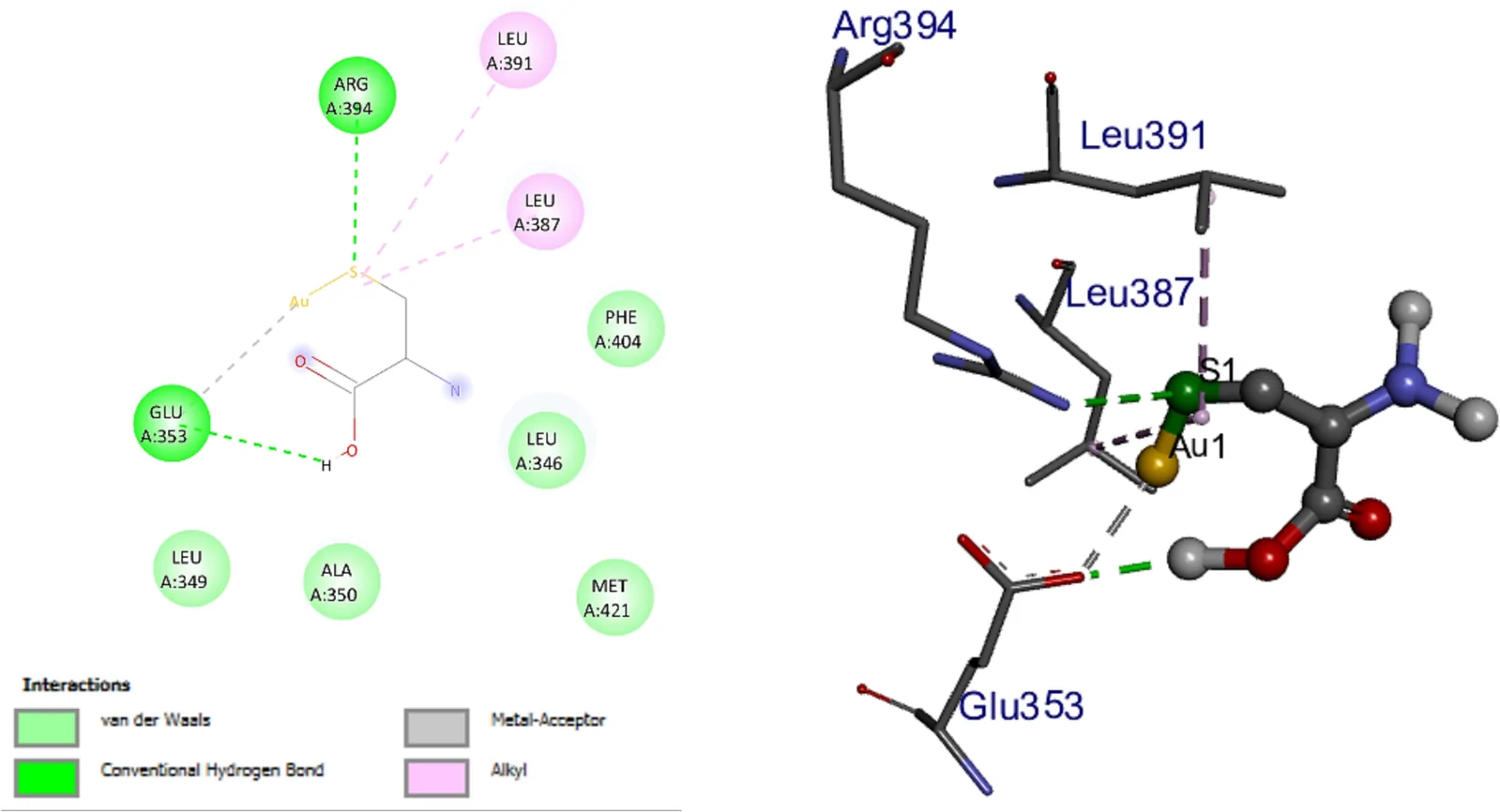
The 2D and 3D interaction analysis of the docked Cys-Au complex within the ERα binding pocket (PDB ID: 3ERT), demonstrating hydrogen-bond, hydrophobic, and Au-associated metal-acceptor interactions with key active-site residues

When the Cys-Au complex was docked against ERα (3ERT), the ligand settled inside the ERα ligand-binding pocket at a binding affinity of − 3.1 kcal/mol. In the chosen pose, hydrogen bonds formed near GLU353 and ARG394, accompanied by hydrophobic contacts at LEU387 and LEU391, and a metal-acceptor interaction linked the Au atom to GLU353 (Fig. 11).

**Fig. 12:**
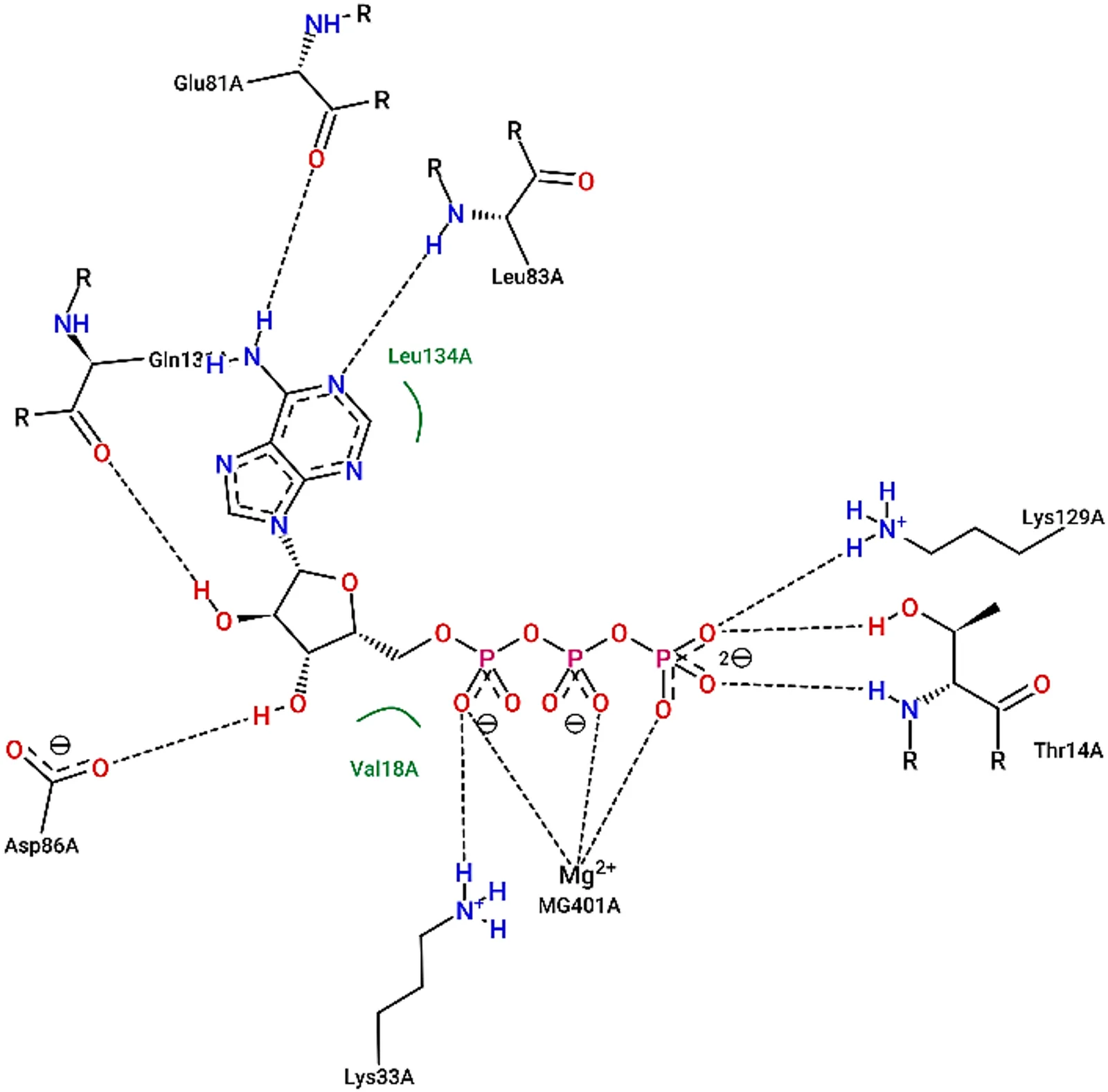
The 2D interaction map of the co-crystallized ATP ligand within the ATP-binding pocket of CDK2 (PDB ID: 1HCK), generated using the ProteinsPlus web server, highlighting key hinge-region and catalytic interactions [15]

For CDK2 (1HCK), the co-crystallized ATP ligand engaged in several stabilizing contacts with LEU83, GLU81, LYS33, ASP86, THR14, and the catalytic Mg^2^⁺ ion, in agreement with the canonical ATP-binding region of CDK2 (Fig. 12).

**Fig. 13:**
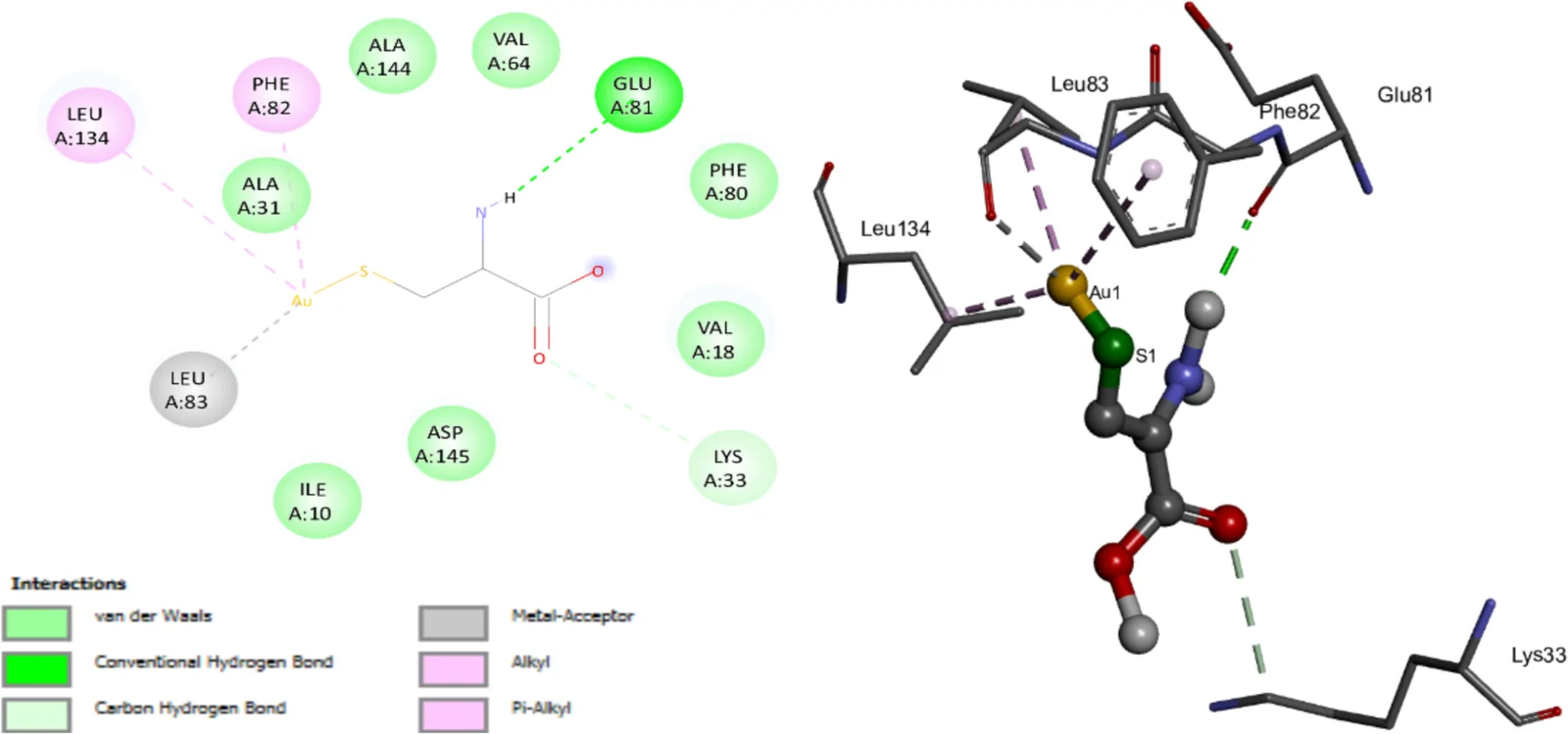
The 2D and 3D interaction analysis of the docked Cys-Au complex within the ATP-binding site of CDK2 (PDB ID: 1HCK), showing hydrogen-bond, hydrophobic, and Au-associated metal-acceptor interactions

Within CDK2, the docked Cys-Au complex sat in the ATP-binding region and contacted residues tied to kinase ligand recognition, among them GLU81, LEU83, LEU134, PHE82, and LYS33. Hydrogen bonding to GLU81 was seen, as were contacts near LEU83, and a metal-acceptor interaction connected the Au atom with LEU83. For the chosen pose the binding affinity was − 3.4 kcal/mol (Fig. 13).

### ADME predictions

**Fig. 14:**
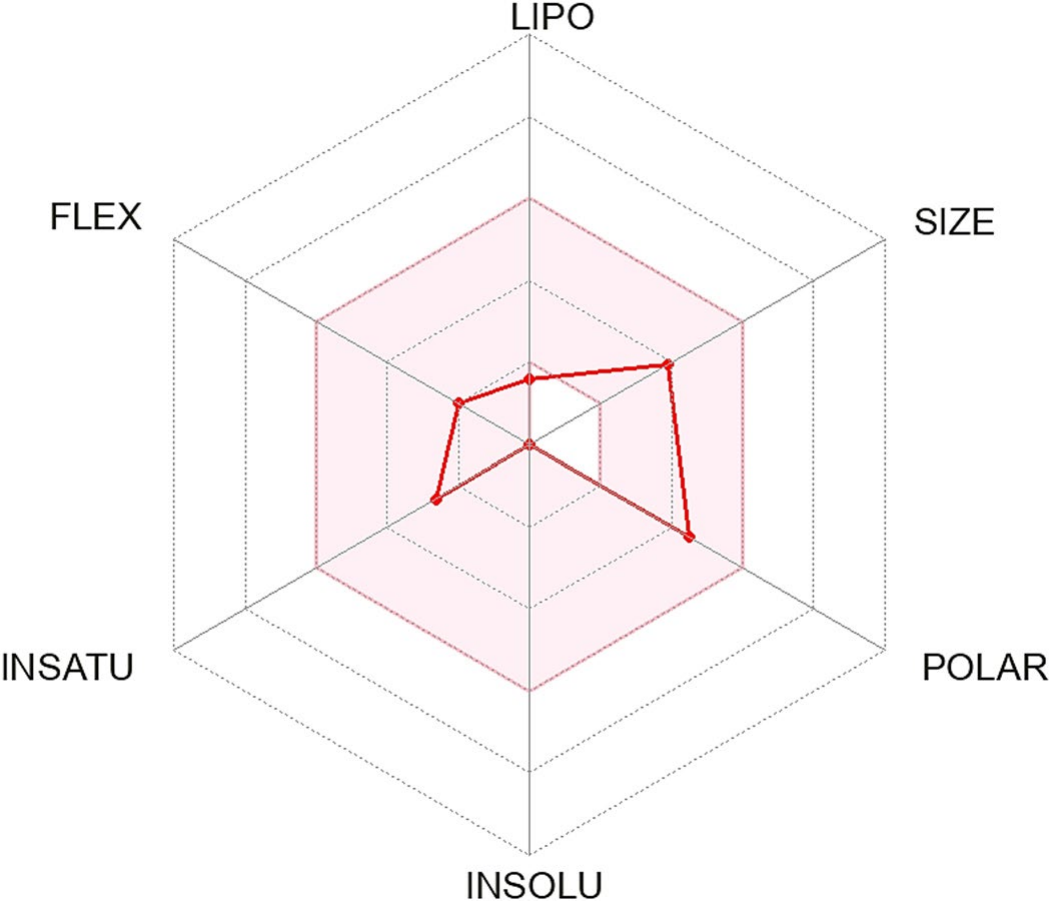
SwissADME bioavailability radar plot, showing predicted physicochemical and drug-likeness properties of the Cys-Au complex [16]

The SwissADME methodology provided the ligand under examination with a unique physicochemical profile (Fig. 14). For the Cys-Au complex, the radar diagram showed low lipophilicity and a restricted range of molecular flexibility.

## Discussion

This study provides evidence that biosynthesized AuNPs possess structural integrity, are biologically active, and inhibit the growth of MCF-7 breast cancer cells. AuNPs were characterized using UV-Vis spectroscopy, Electron Microscopy (TEM), Fourier Transform Infrared Spectroscopy (FTIR), X-Ray Diffraction (XRD), Energy Dispersive X-Ray Spectroscopy (EDX), and Zeta potential. The color transitions through pale yellow, ruby red, and deep purple, along with the maximum SPR at 528 nm, corroborate the expected findings for the formation of colloidal gold. El-Naggar et al. recorded a plasmon band at 540 nm for AuNPs produced by *Streptomyces albogriseolus* [6], Lin et al. documented 525 nm for *Streptomyces* sp. YJD18-mediated AuNPs [7], Aati et al. noted 540 nm for actinomycete-derived AuNPs from *Streptomyces* sp. ASM19 [8], and Soliman et al. placed a peak at 550 nm for biogenic AuNPs derived from cancer-derived metabolites [9], The 22.73–35.61 nm particle size found in the present study likewise carries biological meaning. That range runs somewhat larger than the one obtained by El-Naggar et al., whose particles measured 5.42–13.34 nm from *S. albogriseolus* [6], yet very near the 20–30 nm hydrodynamic range given by Lin et al. for *Streptomyces* sp. YJD18 [7], and similarly close to the 30.4 ± 0.6 nm mean diameter reported by Soliman et al. [9]. The gap relative to the smaller particles of El-Naggar et al. may partly account for why our system displays a different potency pattern and death profile from certain other AuNP reports [6].

Surface charge density provides another informative parameter. While our AuNPs had a positive zeta potential of approximately + 27 mV, a zeta potential of AuNPs derived from YJD18 was reported to be − 11.6 mV, while a zeta potential of AuNPs derived from MCF7 metabolites reported to be − 12.16 ± 1.59 mV [7, 9]. A positive charge may enhance the electrostatic attraction to the negatively charged plasma membrane and may facilitate stronger cellular interaction [31]. The face-centered cubic gold noted in XRD is fully expected and agrees with the work on actinobacterial AuNPs of El-Naggar et al. [6], Aati et al. [8] and Lin et al. [7], and with plant-mediated systems such as Datkhile et al. [32]. Likewise, the FTIR spectrum recorded here shows the presence of a urethane, amine, hydroxyl, and carbohydrate biomolecule layer, which is in line with the heterologous green synthesis concept, in which extracellular metabolites act as both reducing agents and stabilizing agents.

AuNPs generated a clear dose-response relationship against MCF-7 cells across the dose range tested. The corresponding IC_50_ was 8.32 µg/mL, considerably lower than the 18.90 µg/mL obtained for PBMC cells under the same protocol. Compared to the literature, this IC_50_ value falls within the range of biopharmaceutical activity reported for related biogenic AuNP formulations, some more active and some less so. For example, El-Naggar et al. derived IC_50_ values of 22.13 ± 1.3 µg/mL for HepG2 cells for *S. albogriseolus* AuNPs, while an IC_50_ of 3.52 ± 0.1 µg/mL was reported for an AuNPs-doxorubicin conjugate [6]. IC_50_ values of 20.53 µg/mL at 24 h and 12.03 µg/mL at 48 h were reported by Datkhile et al. for Argemone-mediated AuNPs tested on HCT-15 colon cancer cells [32]. Aati et al. reported greater activity with IC_50_ values of 3.77 µg/mL for SCC9 and 1.56 µg/mL for SCC25 from actinomycete-derived AuNPs [8]. The variability in IC_50_ values can be due to the cell line used, NP formulation and NP synthesis method [2] Unlike several of these comparator studies, which report IC50 values for the cancer cell line alone without a matched normal-cell control, the present study measured both MCF-7 and PBMC cytotoxicity under an identical protocol, giving a selectivity index (IC50 PBMC / IC50 MCF-7) of 2.27. A selectivity index above 1 indicates that the biosynthesized AuNPs inhibited MCF-7 cells at a lower concentration than they inhibited normal PBMC cells, consistent with preferential activity against this cancer cell line rather than indiscriminate cytotoxicity, and this matched normal-cell comparison strengthens the antitumor activity documented against MCF-7 itself.

The clearest mechanistic insight derived from this study comes from the analysis of apoptosis. Of the untreated control cells, 95.15% were determined to be viable. Treatment with the AuNPs decreased the viable fraction and increased the population of cells exhibiting early apoptosis at 33.78%, and late apoptosis 16.58%, by a combined total of 50.36%. This observation is of importance, as it confirmed the viability loss observed in the cytotoxicity assay was not due to a suppression of cell metabolism, and instead corresponded to a significant fraction of the cell population undergoing a transition to apoptosis.

Aati et al. [8] found that SCC9 and SCC25 cells exhibited a total of 26.37 and 32.08% apoptosis, respectively, compared to control cells, which showed 0.95–1.95% apoptosis, and early apoptosis fractions of 15.13 and 19.51%. Datkhile et al. [32] found similar results, with AuNP-induced cytotoxicity in HCT-15 cells being linked to greater apoptosis, DNA fragmentation, and increased levels of p53 and caspase-3. Banu et al. [33] also showed that biogenic AuNPs were able to induce apoptosis and increased levels of p53 and decreased Bcl-2 in MCF-7 cells in a dose-dependent manner. For the current formulation, apoptosis was the predominant pathway. However, 39.56% of the cells remained viable, indicating a powerful anticancer effect without complete cellular loss. Doxorubicin, in contrast, caused a more extreme end-point of cellular loss. This type of effect is preferred, as apoptosis is a more regulated pathway of cell death than necrosis.

Analyzing the cell cycle, the untreated cells showed the following profile: 55.21% cells in G0/G1, 34.58% in the S phase, and 10.13% in G2/M. The doxorubicin group showed 51.72% in G0/G1, 39.04% in the S phase, and 9.36% in G2/M, indicating a shift towards the S phase with a marginal decrease in the G0/G1 and G2/M populations.

The AuNPs-treated cells presented that 99.25% of the cells were in G0/G1, with only 0.63% and 0.14% of the population in the S phase and G2/M, respectively. These results suggest that treatment with AuNPs caused a deep G0/G1 phase arrest, which not only prevents cells from entering DNA synthesis but also inhibits the progression to mitosis.

Cell cycle arrest in the G0/G1 phase, which was induced by the AuNPs, also coincided with the decrease in cell viability, a reduction in BrdU incorporation and an increase in the apoptotic fraction. The G0/G1 phase predominance caused by the AuNPs corresponds with the findings of previous studies that show that certain gold nanostructures inhibit cell proliferation by trapping cells in the pre-replicative phase [34–36].

The cell-cycle percentages reported here derive from a single flow-cytometry run per group rather than from multiple biological replicates with a formal statistical comparison, and the full gating strategy used to exclude debris and doublets was not detailed in the Methods; both points limit how precisely the reported percentages should be treated. A complete gating hierarchy, biological replicate number, and an appropriate statistical test (for example, a chi-square or ANOVA on arcsine-transformed phase percentages) is recommended alongside the histograms in follow-up work.

When examining BrdU incorporation, there was a significant decrease from 32.1% at 25 µg/mL to 92.6% at 200 µg/mL. This dose-response was consistently related to the cytotoxicity data and suggest that AuNPs inhibit active proliferation of cells just prior to causing cell lysis. Selim and Hendi showed that there was a concentration-dependent inhibition of cell viability of MCF-7 at 25–200 µg/mL which corresponds with the strong BrdU inhibition at 100–200 µg/mL in this experiment [37].

Reduced BrdU incorporation demonstrates suppression of active DNA synthesis, but it does not by itself establish a discrete S-phase block. Taken together with the cell-cycle data reported above, in which the S-phase fraction was almost completely depleted (0.63%) alongside a near-complete G0/G1 accumulation (99.25%), the more internally consistent interpretation is that AuNP treatment produced a G0/G1 arrest that prevented cells from entering S phase at all, rather than a block acting within an ongoing S phase. Under this interpretation, the marked fall in BrdU incorporation is a downstream consequence of cells being held in G0/G1 and of a parallel loss of viable, cycling cells to apoptosis, rather than direct evidence of S-phase-specific blockade. Throughout this Discussion the phenotype is accordingly described as G0/G1 arrest with secondary suppression of DNA synthesis, rather than as an S-phase block, keeping the cell-cycle and BrdU findings mutually consistent.

Morphologically, the AO/PI images were directionally consistent with this conclusion. Cells that were left untreated were mostly green and showed structural integrity, while the treated cells displayed orange-red to red staining and nuclear condensation. As noted above, however, the magnification used for these images cannot reliably resolve finer structural hallmarks such as shrinkage, membrane blebbing, or discrete apoptotic-body formation, so these specific features are not claimed as directly visualized here; the interpretation instead rests on the AO/PI colour and condensation signal itself, read as qualitative, corroborating evidence alongside the quantitative flow-cytometric apoptosis data. Comparable AO/PI-based morphological evidence of apoptotic death in AuNP-treated MCF-7 cells was reported by Banu et al., who recorded dose-dependent cytotoxicity and apoptotic features in MCF-7 cells exposed to AuNPs [33].

The approximately 65% apoptotic/dead fraction estimated from the AO/PI images was derived from qualitative visual assessment of representative fields rather than from a systematic cell count with a stated number of analysed cells, biological replicates, or an accompanying statistical test. The AO/PI findings are therefore best read as qualitative, corroborating evidence that is directionally consistent with the quantitative Annexin V-FITC/PI flow-cytometry data presented earlier, rather than as an independent quantitative measurement of apoptotic rate in their own right.

In the RT-qPCR data, AuNP treatment cut c-erbB-2 expression to 0.19-fold, an 81% decrease, while p53 rose to 1.70-fold, a 70% increase. This indicates that the AuNPs steered gene expression away from a proliferative oncogenic program and toward a tumor-suppressive program. c-erbB-2 is a major oncogenic driver in breast cancer [38]. whereas p53 ranks among the chief guardians of genomic integrity and a central regulator of cell-cycle arrest and apoptosis [5]. Selim and Hendi found that treating MCF-7 cells with AuNPs markedly upregulated p53 alongside rises in Bax, caspase-3, and caspase-9 and a fall in Bcl-2 [37]. Banu et al. likewise reported upregulation of p53 and downregulation of Bcl-2 in MCF-7 cells following treatment with biosynthesized AuNPs [33].

It is important to be precise about what the RT-qPCR data in the present study do and do not show. A change in c-erbB-2 and p53 mRNA level is evidence of altered gene transcription, but it does not on its own establish altered protein abundance, activity, or downstream pathway output; we have therefore avoided describing this as functional pathway modulation and instead describe it as transcriptional evidence consistent with a shift away from a proliferative, oncogenic expression program and toward a tumor-suppressive one. Direct confirmation at the protein level, for example by western blot for c-erbB-2 and p53 together with downstream effectors such as p21, Bax, Bcl-2, and the caspases, together with appropriate biological replication and statistical testing, was not performed in the present study and is identified as a specific, high-priority direction for follow-up work in the Conclusion.

The docking results showed that the simplified Cys–Au corona model could establish plausible interactions within biologically relevant regions of ERα and CDK2, providing complementary mechanistic insight into possible protein–corona interactions associated with the biosynthesized AuNPs. With ERα, the Cys-Au complex formed a metal-acceptor interaction between the Au atom and GLU353. That contact carries weight because GLU353 is one of the key residues anchoring ligands in ERα. These interactions should be interpreted qualitatively, as the Cys–Au complex represents only a simplified molecular motif of the heterogeneous biological corona rather than the complete nanoparticle surface. The comparatively modest binding affinity (− 3.1 kcal/mol) was anticipated because the Cys–Au complex represents only a small fragment of the heterogeneous biological corona. FTIR analysis demonstrated the presence of additional peptide-, amide-, and carbohydrate-associated functional groups on the AuNP surface, suggesting that the native biological corona is considerably more complex and capable of establishing additional multivalent interactions that are not captured by the simplified docking model.

In CDK2, the ATP-binding pocket displayed the anticipated hinge-region contacts at LEU83 along with catalytic interactions tied to GLU81, ASP86, and Mg2⁺ coordination. Both ligands studied settled in regions overlapping the ATP-binding cavity. For the Cys-Au complex, a metal-acceptor interaction involving Au and LEU83 accompanied hydrogen bonding with GLU81. Since LEU83 is an important hinge-region residue for many CDK2 inhibitors, this observation suggests that sulfur-containing biomolecular motifs associated with the AuNP corona may establish localized interactions within the kinase active site. However, these findings should be regarded as qualitative structural observations rather than evidence of strong target engagement.

The calculated binding affinities for the Cys-Au complex against ERα (-3.1 kcal/mol) and CDK2 (-3.4 kcal/mol) are, in absolute terms, weak by the standards of a purpose-designed small-molecule inhibitor and are not intended to be read as strong, independent evidence of specific target engagement. The docking exercise instead served a narrower, hypothesis-generating purpose: because the true biomolecular corona of the biosynthesized AuNPs is a complex and only partially characterized mixture of proteins, polysaccharides, and secondary metabolites, the cysteine-gold complex was chosen as a deliberately simplified, tractable surrogate for the sulfur- and amide-linked interactions most likely to occur at the corona-target interface, rather than as a literal model of any single corona component. The modest affinities obtained are consistent with a surface-level, corona-mediated stabilizing contact rather than with high-affinity, drug-like binding. The docking results are accordingly described throughout this section as correlative and hypothesis-generating, requiring direct biochemical confirmation, rather than as demonstrated evidence of target engagement.

The CDK2 docking result is, in our view, the more directly relevant of the two findings to the pronounced G0/G1 arrest observed by flow cytometry. CDK2, in complex with cyclin E, is the principal kinase responsible for phosphorylating the retinoblastoma protein (Rb) at the G1/S checkpoint; Rb phosphorylation releases E2F transcription factors and licenses entry into S phase. Interference with CDK2 activity, or with the hinge-region and ATP-pocket interactions engaged by the docked Cys-Au complex (contacts with GLU81 and the hinge residue LEU83), would be expected to leave Rb in its hypophosphorylated, E2F-bound state, thereby blocking the G1-to-S transition. This is consistent with, and offers a plausible molecular explanation for, the near-complete G0/G1 arrest observed experimentally, in which 99.25% of AuNP-treated cells accumulated in G0/G1 while the S and G2/M fractions were almost entirely depleted. ERα engagement, by contrast, is better regarded as a complementary, parallel mechanism relevant to the estrogen-receptor-driven proliferative and survival signaling of MCF-7 cells, rather than as a direct driver of the cell-cycle-arrest phenotype itself. It should be emphasized that the Cys-Au complex is a simplified surrogate for the sulfur- and amide-linked interactions present within the full biomolecular corona of the biosynthesized AuNPs, and that the docking results are therefore correlative and hypothesis-generating; direct biochemical assays of CDK2 kinase activity and Rb phosphorylation status in AuNP-treated cells represent an important direction for future confirmatory work.

## Conclusion

The study reveals that biosynthesized AuNPs possessed the physicochemical characteristics of stable nanoscale gold, and in addition, the NPs demonstrated a robust and concentration-dependent inhibitory response against MCF-7 breast cancer cells. The AuNPs exhibited the ability to reduce viability, inhibit proliferation, induce cell death along with significant apoptotic alterations, and disrupt the cell cycle. In addition, the NPs caused a down regulation of the c-erbB-2 oncogene with an up regulation of the p53 tumor suppressor. These alterations justify the mechanism that combines an anti-cancer effect with the arrest of cell growth and the tumor-suppressive pathways with a synergistic effect. Collectively, the results of these studies suggest that the biosynthesized AuNPs demonstrate a multi-targeted strategy to inhibit the progression of human breast cancer by means of inhibiting cell growth, apoptosis, and the remodeling of key gene expressions.

## Supplementary Information

Below is the link to the electronic supplementary material.

Supplementary Material 1.

## Data Availability

No datasets were generated or analysed during the current study.

## Abbreviations

AuNPs: Gold nanoparticles
SPR: Surface plasmon resonance
FTIR: Fourier transform infrared spectroscopy
XRD: X-ray diffraction
EDX: Energy-dispersive X-ray spectroscopy
TEM: Transmission electron microscopy
PBMC: Peripheral blood mononuclear cells
MTT: 3-(4,5-dimethylthiazol-2-yl)-2,5-diphenyltetrazolium bromide
DMEM: Dulbecco’s Modified Eagle Medium
FBS: Fetal bovine serum
RT-qPCR: Reverse transcription quantitative polymerase chain reaction
cDNA: Complementary DNA
GAPDH: Glyceraldehyde-3-phosphate dehydrogenase
BrdU: 5-bromo-2′-deoxyuridine
CLSM: Confocal laser scanning microscopy
AO/PI: Acridine orange/propidium iodide
ADME: absorption distribution metabolism and excretion
ERα: Estrogen receptor alpha
CDK2: Cyclin-dependent kinase 2
PDB: Protein Data Bank
IC50: Half-maximal inhibitory concentration
ANOVA: Analysis of variance
HSD: Honestly significant difference
SD: Standard deviation
SE: Standard error
CV: Coefficient of variation
