# Supplementary material for: Employing Streptomyces enissocaesilis as a sustainable cell factory for green synthesis of gold nanoparticles with multi-target anticancer activity against MCF-7 breast cancer cells
Source: Microb Cell Fact. 2026 Sep 28;25:193. doi: 10.1186/s12934-026-03113-8 (PMC13625260; doi:10.1186/s12934-026-03113-8)
Supplement: Supplementary file 1 [file 12934_2026_3113_MOESM1_ESM.docx]

**Table S1.** Primer sequences.

| **Gene** | **Primer sequence** |
| --- | --- |
| **GAPDH (NM_002046.7)** | Forward: 5'-ACAACTTTGGTATCGTGGAAGG-3' |
|  | Reverse: 5'-GCCATCACGCCACAGTTTC-3' |
| **c-erbB-2 (HER2/ERBB2; NM_004448.4)** | Forward: 5'-TGTGCAGATCATGGAGGAAG-3' |
|  | Reverse: 5'-CTGGACATCTTCTTCCATCTCTT-3' |
| **p53 (TP53; NM_000546.6)** | Forward: 5'-CCTCAGCATCTTATCCGAGTGG-3' |
|  | Reverse: 5'-TGGATGGTGGTACAGTCAGAGC-3' |

**Table S2.** Physical characterization summary of the biogenic synthesized AuNPs.

| **Parameter** | **Observed finding** | **Interpretation** |
| --- | --- | --- |
| **Visual appearance** | Pale yellow to ruby red/deep purple | Macroscopic evidence of AuNP formation by SPR |
| **UV-Vis** | Major peak at 528 nm; secondary band ~430 nm | Gold plasmon band plus metabolite/corona contribution |
| **TEM morphology** | Quasi-spherical to ellipsoidal particles | Biogenic nanoscale gold structures |
| **Size range** | 22.73-35.61 nm | Within bioactive low tens-of-nanometers range |
| **Zeta potential** | +27 mV | Moderate-to-good electrosteric stability |
| **XRD** | FCC Au with (111), (200), (220), (311) reflections | Crystalline metallic gold |
| **EDX** | Gold signal with oxygen-rich bio-organic matrix | Successful reduction plus organic capping |

**Table S3.** The *in vitro* safety profile of the biogenically synthesized AuNPs on normal PBMC cells. Rep 1-3 are technical replicate wells within a single experiment, not independent biological replicates.

| **Dose (µg/mL)** | **Rep 1** | **Rep 2** | **Rep 3** | **Mean absorbance** | **SD** | **95% CI** | **Inhibition (%)** | **CV (%)** |
| --- | --- | --- | --- | --- | --- | --- | --- | --- |
| 3.125 | 0.5980 | 0.6240 | 0.6155 | 0.6125 | 0.0133 | 0.5796 to 0.6454 | 12.50 | 2.16 |
| 6.25 | 0.5220 | 0.5470 | 0.5312 | 0.5334 | 0.0126 | 0.5020 to 0.5648 | 23.80 | 2.37 |
| 12.5 | 0.4280 | 0.4570 | 0.4380 | 0.4410 | 0.0147 | 0.4044 to 0.4776 | 37.00 | 3.34 |
| 25 | 0.2490 | 0.2760 | 0.2648 | 0.2633 | 0.0136 | 0.2296 to 0.2970 | 62.39 | 5.15 |
| 50 | 0.0680 | 0.0890 | 0.0824 | 0.0798 | 0.0107 | 0.0531 to 0.1065 | 88.60 | 13.46 |
| 100 | 0.0170 | 0.0220 | 0.0198 | 0.0196 | 0.0025 | 0.0134 to 0.0258 | 97.20 | 12.79 |

**Table S4.** Statistical analysis summary of the safety profile results of biogenically synthesized AuNPs on normal PBMC cells.

| **Statistical metric** | **Result** |
| --- | --- |
| **One-way ANOVA** | F = 1244.7; p = 7.43 × 10^-16 |
| **Log-dose vs inhibition regression** | r = 0.988; p = 0.00021 |
| **Estimated IC_50_** | 18.90 µg/mL |
| **Most variable concentration** | 50 µg/mL (CV = 13.46%) |
| **Lowest variability** | 3.125 µg/mL (CV = 2.16%) |

**Table S5.** The *in vitro* cytotoxic activity of the biogenically synthesized AuNPs on MCF-7 breast cancer cells. Rep 1-3 are technical replicate wells within a single experiment, not independent biological replicates.

| **Dose (µg/mL)** | **Rep 1** | **Rep 2** | **Rep 3** | **Mean absorbance** | **SD** | **95% CI** | **Inhibition (%)** | **CV (%)** |
| --- | --- | --- | --- | --- | --- | --- | --- | --- |
| 3.125 | 0.552 | 0.566 | 0.559 | 0.5590 | 0.0070 | 0.5416 to 0.5764 | 20.14 | 1.25 |
| 6.25 | 0.414 | 0.398 | 0.407 | 0.4063 | 0.0080 | 0.3864 to 0.4263 | 41.95 | 1.97 |
| 12.5 | 0.236 | 0.221 | 0.229 | 0.2287 | 0.0075 | 0.2100 to 0.2473 | 67.33 | 3.28 |
| 25 | 0.112 | 0.101 | 0.106 | 0.1063 | 0.0055 | 0.0927 to 0.1200 | 84.81 | 5.18 |
| 50 | 0.041 | 0.033 | 0.036 | 0.0367 | 0.0040 | 0.0266 to 0.0467 | 94.76 | 11.02 |
| 100 | 0.013 | 0.010 | 0.012 | 0.0117 | 0.0015 | 0.0079 to 0.0155 | 98.33 | 13.09 |

**Table S6.** Statistical analysis summary of the cytotoxicity results of biogenically synthesized AuNPs on MCF-7 breast cancer cells.

| **Statistical metric** | **Result** |
| --- | --- |
| **One-way ANOVA** | F = 3943.2; p = 7.42 × 10^-19 |
| **Log-dose vs inhibition regression** | r = 0.969; p = 0.00142 |
| **Estimated IC_50_** | 8.32 µg/mL |
| **Most variable concentration** | 100 µg/mL (CV = 13.09%) |
| **Lowest variability** | 3.125 µg/mL (CV = 1.25%) |

## Table S7. Cell-cycle distribution in the control non-treated, doxorubicin-treated, and AuNPs-treated cells

| **Cells** | **G0/G1 (%)** | **S phase (%)** | **G2/M (%)** |
| --- | --- | --- | --- |
| **Control non-treated** | 55.21 | 34.58 | 10.13 |
| **Doxorubicin-treated** | 51.72 | 39.04 | 9.36 |
| **AuNPs-treated** | 99.25 | 0.63 | 0.14 |

**Table S8.** BrdU incorporation and proliferation inhibition in AuNPs-treated MCF-7 cells (48 h)

| **AuNPs concentration (µg/mL)** | **Absorbance (450 nm, mean ± SD)** | **% Proliferation inhibition** |
| --- | --- | --- |
| 0 (Control) | 1.250 ± 0.045 | 0 |
| 25 | 0.848 ± 0.032 | 32.1 ± 2.6 |
| 50 | 0.541 ± 0.028 | 56.7 ± 2.2 |
| 100 | 0.269 ± 0.019 | 78.4 ± 3.2 |
| 200 | 0.092 ± 0.012 | 92.6 ± 1.0 |

**Table S9.** Relative expression of **c-erbB-2** and **p53** in untreated and AuNPs-treated cells measured by RT-qPCR. Expression values were normalized to the housekeeping gene, and untreated cells were used as the calibrator group.

| **Gene** | **Functional role** | **Untreated cells (relative expression)** | **AuNP-treated cells (relative expression)** | **Change vs untreated** | **Regulation** |
| --- | --- | --- | --- | --- | --- |
| c-erbB-2 | Oncogene | 1.00 | 0.19 | −81% | Downregulated |
| p53 | Tumor suppressor gene | 1.00 | 1.70 | +70% | Upregulated |
